# Bioinformatics-based analysis of the relationship between disulfidptosis and prognosis and treatment response in pancreatic cancer

**DOI:** 10.1038/s41598-023-49752-4

**Published:** 2023-12-14

**Authors:** Yuanpeng Xiong, Xiaoyu Kong, Haoran Mei, Jie Wang, Shifa Zhou

**Affiliations:** 1 Department of General Surgery, The First Affiliated Hospital of Nanchang University, Nanchang, 330006 Jiangxi China; 2 School of Public Health, Nanchang University, Nanchang, 330006 Jiangxi China; 3 Department of Emergency Surgery, The First Affiliated Hospital of Nanchang University, Nanchang, 330006 Jiangxi China

**Keywords:** Cancer, Computational biology and bioinformatics, Genetics, Immunology

## Abstract

Tumor formation is closely associated with disulfidptosis, a new form of cell death induced by disulfide stress-induced. The exact mechanism of action of disulfidptosis in pancreatic cancer (PCa) is not clear. This study analyzed the impact of disulfidptosis-related genes (DRGs) on the prognosis of PCa and identified clusters of DRGs, and based on this, a risk score (RS) signature was developed to assess the impact of RS on the prognosis, immune and chemotherapeutic response of PCa patients. Based on transcriptomic data and clinical information from PCa tissue and normal pancreatic tissue samples obtained from the TCGA and GTEx databases, differentially expressed and differentially surviving DRGs in PCa were identified from among 15 DRGs. Two DRGs clusters were identified by consensus clustering by merging the PCa samples in the GSE183795 dataset. Analysis of DRGs clusters about the PCa tumor microenvironment and differential analysis to obtain differential genes between the two DRG clusters. Patients were then randomized into the training and testing sets, and a prognostic prediction signature associated with disulfidptosis was constructed in the training set. Then all samples were divided into high-disulfidptosis-risk (HDR) and low-disulfidptosis-risk (LDR) subgroups based on the RS calculated from the signature. The predictive efficacy of the signature was assessed by survival analysis, nomograms, correlation analysis of clinicopathological characteristics, and the receiver operating characteristic (ROC) curves. To assess differences between different risk subgroups in immune cell infiltration, expression of immune checkpoint molecules, somatic gene mutations, and effectiveness of immunotherapy and chemotherapy. The GSE57495 dataset was used as external validation, reverse transcription-quantitative polymerase chain reaction (RT-qPCR) was used to detect the expression levels of DRGs. A total of 12 DRGs with differential expression and prognosis in PCa were identified, based on which a risk-prognosis signature containing five differentially expressed genes (DEGs) was developed. The signature was a good predictor and an independent risk factor. The nomogram and calibration curve shows the signature's excellent clinical applicability. Functional enrichment analysis showed that RS was associated with tumor and immune-related pathways. RS was strongly associated with the tumor microenvironment, and analysis of response to immunotherapy and chemotherapy suggests that the signature can be used to assess the sensitivity of treatments. External validation further demonstrated the model's efficacy in predicting the prognosis of PCa patients, with RT-qPCR and immunohistochemical maps visualizing the expression of each gene in PCa cell lines and the tissue. Our study is the first to apply the subtyping model of disulfidptosis to PCa and construct a signature based on the disulfidptosis subtype, which can provide an accurate assessment of prognosis, immunotherapy, and chemotherapy response in PCa patients, providing new targets and directions for the prognosis and treatment of PCa.

## Introduction

Pancreatic cancer (PCa) is one of the few malignancies with a similar incidence and mortality rate and ranks seventh in cancer mortality worldwide^1^. PCa has made some progress in recent years with surgery and comprehensive treatment, but as the clinical symptoms of PCa are not obvious in the early stage and the disease progresses very rapidly, most patients have already developed local progression or distal metastasis at the time they are detected, thus forfeiting the opportunity for surgery. Even when patients undergo an early surgical resection, more than 90 percent experience a recurrence^2^, so their overall 5-year survival remains unsatisfactory at only about 10%^3^. Therefore, continuous research into the pathophysiological mechanisms of PCa and the exploration of new and effective therapeutic targets are essential to improve the prognosis of patients with PCa.

Regulated cell death (RCD) and accidental cell death (ACD) are the two main classifications of cell death^4^. Among these, RCD also referred to as programmed cell death, can be divided into noninflammatory (apoptosis) and inflammatory (regulatory necrosis). There are various forms of regulated necrosis, including pyroptosis, autophagy, necrotizing apoptosis, and ferroptosis, among others^5^. Regulated necrosis is a well-known factor in the onset and growth of tumors^6^. This is also true in PCa, for example, it has been shown that the induction of pyroptosis can inhibit PCa progression^7^, autophagy can promote the immune escape of PCa cells^8^, and PCa cells by inhibiting the onset of ferroptosis thus promoting proliferation, migration, and invasion^9^.

Disulfidptosis is a completely new mode of cell death stated by Liu et al^10^. Their study found that cancer cells with high SLC7A11 expression under glucose deficient experience an abnormal accumulation of intracellular disulfide-like molecules such as cysteine, which induces disulfide stress that raises the disulfide bond content in the actin cytoskeleton, causing the actin filaments to contract and the cytoskeletal structure to collapse, resulting in rapid cell death. This mode of cell death differs from known apoptosis, ferroptosis, and many others. Furthermore, the role of disulfidptosis in PCa is unknown. Thus, disulfidptosis may open up new avenues for tumor therapy, but further studies are needed to fully understand its unique process and potential therapeutic use.

In this study, we identified two subtypes of disulfidptosis by identifying different clustering features and investigated their association with survival and immune infiltration in PCa patients, then constructed a risk score for disulfidptosis by typing differentially expressed genes and further analyzed its value in assessing the diagnosis, prognosis, tumor immune infiltration and response to immunotherapy and chemotherapy in PCa patients.

## Methods

### Data acquisition

Patient data were obtained from the TCGA database (https://portal.gdc.cancer.gov/) (project ID, TCGA-PAAD) containing data from 178 PCa samples and 4 normal pancreas samples. The 167 normal pancreatic samples from the GTEx database (https://commonfund.nih.gov/GTEx) and 134 PCa samples containing survival data from GSE183795 in the GEO database (https://www.ncbi.nlm.nih.gov/geo/) were included to expand the sample size. A sample of 63 PCa cases was also extracted from the GSE57495 dataset as an external validation dataset (Supplementary Table S1). Extract the complete RNA-seq data of the samples from these three databases. The mutation data from the TCGA samples were also downloaded. Format normalization of patient gene expression data from several data sources using the 'SVA' R package.

### Certification of disulfidptosis-related genes (DRGs) in PCa

In total, we obtained 15 DRGs from Liu's study, including FLNA, FLNB, MYH9, TLN1, ACTB, MYL6, MYH10, CAPZB, DSTN, IQGAP1, ACTN4, PDLIM1, CD2AP, INF2, and SLC7A11(the gene set consists of the key gene for disulfidptosis, SLC7A11, and the genes most strongly associated with disulfidptosis). The expression and copy number variation (CNV) data of these 15 DRGs were extracted and the frequency of CNV was calculated based on the proportion of amplifications and deletions in CNV. Differential analysis of DRGs and Kaplan–Meier survival analysis were performed using the “limma” R package and the “survivor” R package. 12 DRGs with both expression and survival differences in PCa were identified by taking the intersection using the “VennDiagram” R package.

### Consensus clustering analysis for DRGs

Clustering analyses were performed using the “ConensusClusterPlus” R package, optimal k-clusters of DRGs were constructed by selecting k-values with high cluster stability based on the optimal clustering criteria. The optimal clustering criteria are as follows: the sample size of any group should not be too small; a decrease in the downward slope is observed in the cumulative distribution function (CDF) curve; and the intra-cluster correlation increases and the inter-cluster correlation decreases after clustering. Survival analysis was performed with the “survival” R package. Heat mapping using the "pheatmap" R package to analyze differences in clinical characteristics between clusters. The distribution of the samples among the different clusters was analyzed and plotted using the PCA algorithm and the “ggplot2” R package.

### Evaluation of the tumor microenvironment (TME) by clustered subtypes

The "ESTIMATE" R package was used to detect changes in TME in PCa patients with different subtypes of PCa to determine if there was an association between the subtypes obtained by clustering and TME. The “GSEABase” and the “GSVA” R packages were also used to assess the enrichment differences between the 23 human immune cell subtypes.

### Identification of differentially expressed genes (DEGs) of the disulfidptosis subtypes and the establishment of a prognostic signature

Using the “limma” R package, DEGs of the disulfidptosis subtypes were identified. Based on adjusted *p* < 0.001 and |log2FC|> 1, we identified DEGs between clusters. A disulfidptosis-related prognostic signature was developed to statistically evaluate the correlation between the disulfidptosis pattern and PCa. Genes associated with survival were screened in DEGs using univariate Cox analysis. Subsequently, we constructed the prognostic risk signature associated with disulfidptosis using Lasso and multivariate Cox (Lasso–Cox) regression analysis. All PCa samples were divided into a training cohort (n = 155) and a test cohort (n = 157) on a 1:1 ratio. The risk scores (RS) of the samples were calculated according to the model equations. All PCa patients participating in the study were defined as high-disulfidptosis-risk (HDR) and low-disulfidptosis-risk (LDR) subgroups according to common risk score classification rules (median method). Kaplan–Meier survival analysis was used to evaluate the significance of RS in clinical prognosis. Receiver operating characteristic (ROC) curves were used to verify the accuracy of the signature predictions. The association between RS and subtypes was demonstrated using the “ggalluvial” R package.

### Construction and verification of nomogram

To determine the prognosis of PCa, we created a nomogram based on RS associated with disulfidptosis. Multiple clinical characteristics and RS of PCa patients were used to create a nomogram, and the corresponding 1-, 3-, and 5-year survival probabilities were calculated based on the patients’ different scores. This nomogram was developed with the help of the "RMS" R package. The Nomogram's predictions were evaluated for accuracy using a calibration curve.

### Functional enrichment analysis of disulfidptosis-related genes

To explore the functional potential between different subtypes and risk subgroups, we performed GSEA for Kyoto Encyclopedia of Genes and Genomes (GSEA-KEGG)^11,12^ functional enrichment analysis of differential genes between different risk subgroups.

### Relationship between TME and immune status in risk subgroups

The CIBERSORT algorithm was used to further analyze the infiltration of immune cells in the disulfidptosis risk subgroups. Not only were differences in immune checkpoint genes between risk subgroups compared but IPS scores were also obtained from The Cancer Immunome Atlas (TCIA; https://tcia.at/home) to assess the efficacy of treatment with immune checkpoint inhibitors (ICIs) by comparing IPS between risk subgroups. The "ggpubr" R package was used to do the analysis.

### Tumor mutation burden (TMB) and drug sensitivity analysis

TMB creates novel immunogenicity and was previously assumed to predict the effectiveness of immune checkpoint blockade therapy^13^. Mutation profiles of two risk subgroups were performed using the “MAFTOOLS” R package to visualize the frequency and type of mutant genes. To graphically depict the differences between two risk subgroups on the TMB, we employed violin plots. The "pRophetic" R package was used to determine the half-inhibitory concentrations (IC50) of 138 chemotherapeutic agents. In addition, the Wilcoxon signed-rank test was used to compare the different risk subgroups.

### External validation

The GEO dataset GSE57495 was used to extract the expression of DRGs in the risk model and plot Kaplan–Meier survival curves and ROC curves to further serve as external validation.

### Quantitative reverse transcription PCR(RT-qPCR) and immunohistochemical analysis

In this study, the researchers acquired the hTERT-HPNE human pancreatic duct epithelial cell line along with the PANC-1 and AsPC-1 human PCa cell lines from the esteemed Chinese Academy of Sciences type culture collection. Total cellular RNA was extracted according to the operating details of the RNAsimple Total RNA Kit (Tiangen Biotech). Total RNA was reverse transcribed to cDNA using the FastKing RT kit (Tiangen Biotech). SuperReal PreMix Plus (SYBR Green) was added in a real-time quantitative polymerase chain reaction. Primer sequences used in the assay are shown in Supplementary Table S2. Immunohistochemical data of typed genes and genes in the risk model were extracted from The Human Protein Atlas (https://www.proteinatlas.org/), and their expression in tissues was demonstrated concretely.

### Statistical analyses

Statistical analysis using R version 4.2.2 and SPSS25. The log-ranch test was used for survival analysis. The Wilcoxon test was used for the analysis of differences between groups. Spearman analysis was used for correlation analysis. Clinical characteristics were analyzed using the Chi-square test or Fisher’s exact test. Differences between groups were statistically significant (*p* < 0.05).

## Results

### Identification of DRGs in PCa

**Figure 1:**
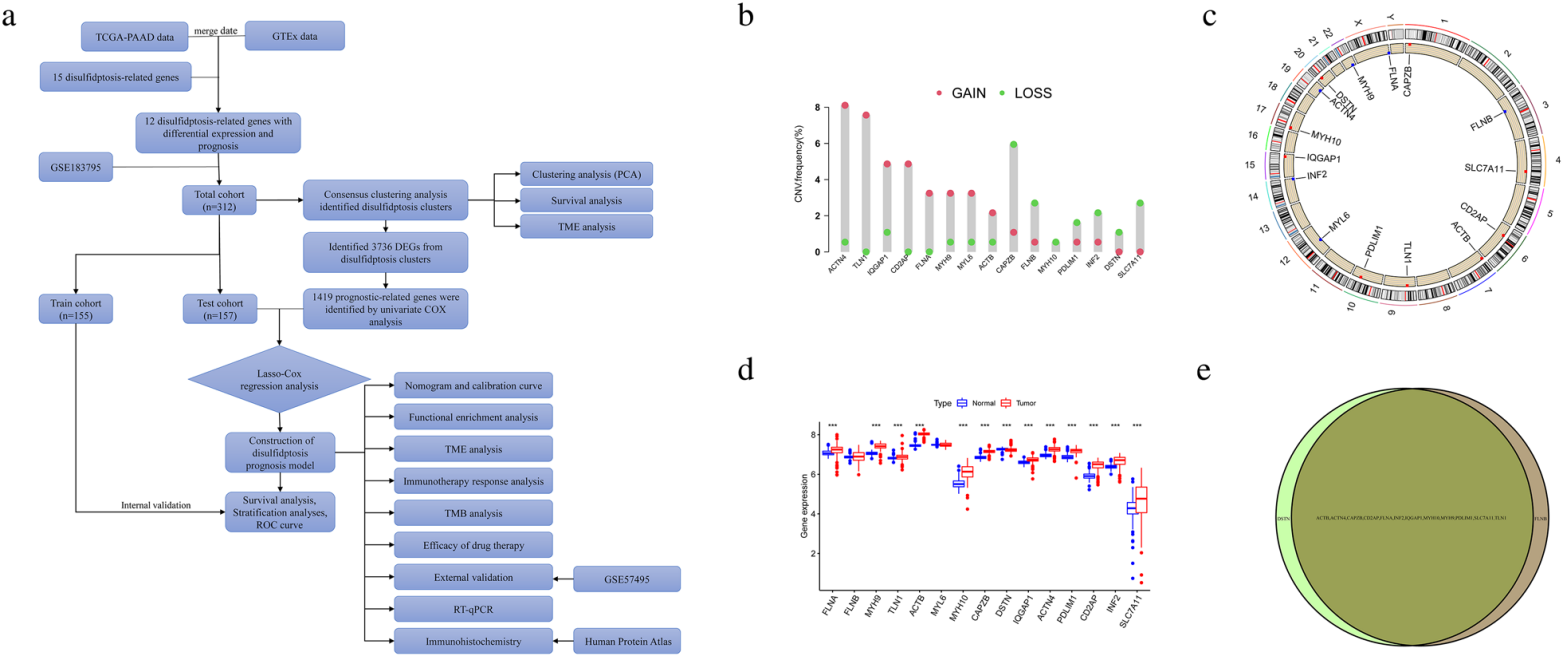
Study flow chart and expression and analysis of 15 DRGs in pancreatic cancer (**a**) the flow chart of this study. (**b**) The frequency of CNV of DRGs in 178 pancreatic cancer patients in TCGA-PAAD. (**c**) distribution of the 15 DRGs on the chromosomes of pancreatic cancer patients, with scattered red dots representing amplification frequency and scattered blue dots representing deletion frequency. (**d**) differential expression of 15 DRGs in pancreatic cancer tissues and normal control pancreatic tissues. (**e**) the intersection was taken to obtain 12 DRGs with expression differences and survival differences. *p < 0.05, **p < 0.01, ***p < 0.001.

Figure 1a showed the flow chart of this study. To explore the role of DRGs in PCa, we analyzed the gene expression profiles of these 15 DRGs in PCa patients. As shown in Fig. 1b, for ACTN4, TLN1, IQGAP1, CD2AP, FLNA, MYH9, MYL6, and ACTB genes, the CNV amplification frequency was greater than the CNV deletion frequency in PCa patients. In contrast, for the CAPZB, FLNB, MYH10, PDLIM1, INF2, DSTN, and SLC7A11 genes, the CNV deletion frequency was greater than the CNV amplification frequency in PCa patients. Also, the location of the 15 DRGs on the chromosomes of PCa patients was shown in Fig. 1c. Normal pancreatic samples from the GTEx database were included to further compare whether these 15 DRGs were differentially expressed between PCa tissue and normal pancreatic tissue. The results are shown in Fig. 1d, except for FLNB and MYL6, the remaining 13 DRGs were significantly differentially expressed in the normal and tumor groups of the pancreas. To further explore the presence of PCa prognosis-related genes in the 15 DRGs, we combined TCGA-PAAD and GSE183795 to further expand the PCa sample data. Results Supplementary Figure S1 showed that all 13 DRGs except DSTN and MYL6 were statistically significant in terms of differences in survival rates for patients with PCa. We then used the Venn diagram to identify 12 DRGs with differential expression and differential survival (Fig. 1e), which play an important role in the prognosis of PCa patients.

### Consensus clustering analysis of DRGs

**Figure 2:**
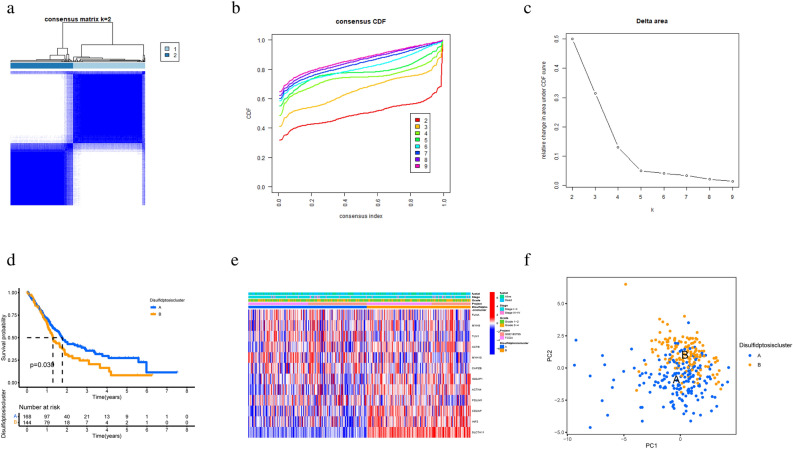
Consensus clustering analysis based on 12 DRGs (**a**–**c**) consensus matrix results graph (k = 2). (**a**) The consensus clustering matrix for k = 2. (**b**) Cumulative distribution function (CDF) with the k value from 2 to 9. (**c**) Relative change in area under the CDF curve according to different k values. (**d**) Kaplan–Meier survival curves showed a statistically significant difference between the two subtypes (*p* = 0.030). (**e**) heat map of clinical features between the two subtypes. (**f**) PCA analysis of pancreatic cancer samples between two subtypes.

To explore the expression characteristics of the 12 DRGs identified in PCa, a total of 312 PCa samples from the TCCA-PAAD and GSE183795 cohorts were clustered using a consensus clustering algorithm based on the expression data of these 12 DRGs. Based on the results of the cluster analysis shown in Fig. 2a–c, the cluster had the best stability when k = 2, and thus all patients were divided into two subgroups (subtype A and subtype B), with subtype A containing 168 samples and subtype B containing 144 samples. Further Kaplan–Meier survival curves were plotted between cluster A and cluster B to compare whether there was a survival difference. The results are shown in Fig. 2d. The prognosis of patients with PCa in cluster A was significantly better than in cluster B (*p* = 0.030). A heat map based on these 12 DRGs was drawn based on clinical characteristics shared between the TCCA-PAAD and GSE183795 datasets (Fig. 2e), suggesting that subtype B expresses these 12 DRGs more frequently than subtype A. The PCA principal component analysis further showed that the subtypes obtained from clustering based on these 12 DRGs could differentiate PCa patients, with patients with different subtypes of PCa patients being assigned to different regions (Fig. 2f).

### Analysis of the TME based on two disulfidptosis subtypes

**Figure 3:**
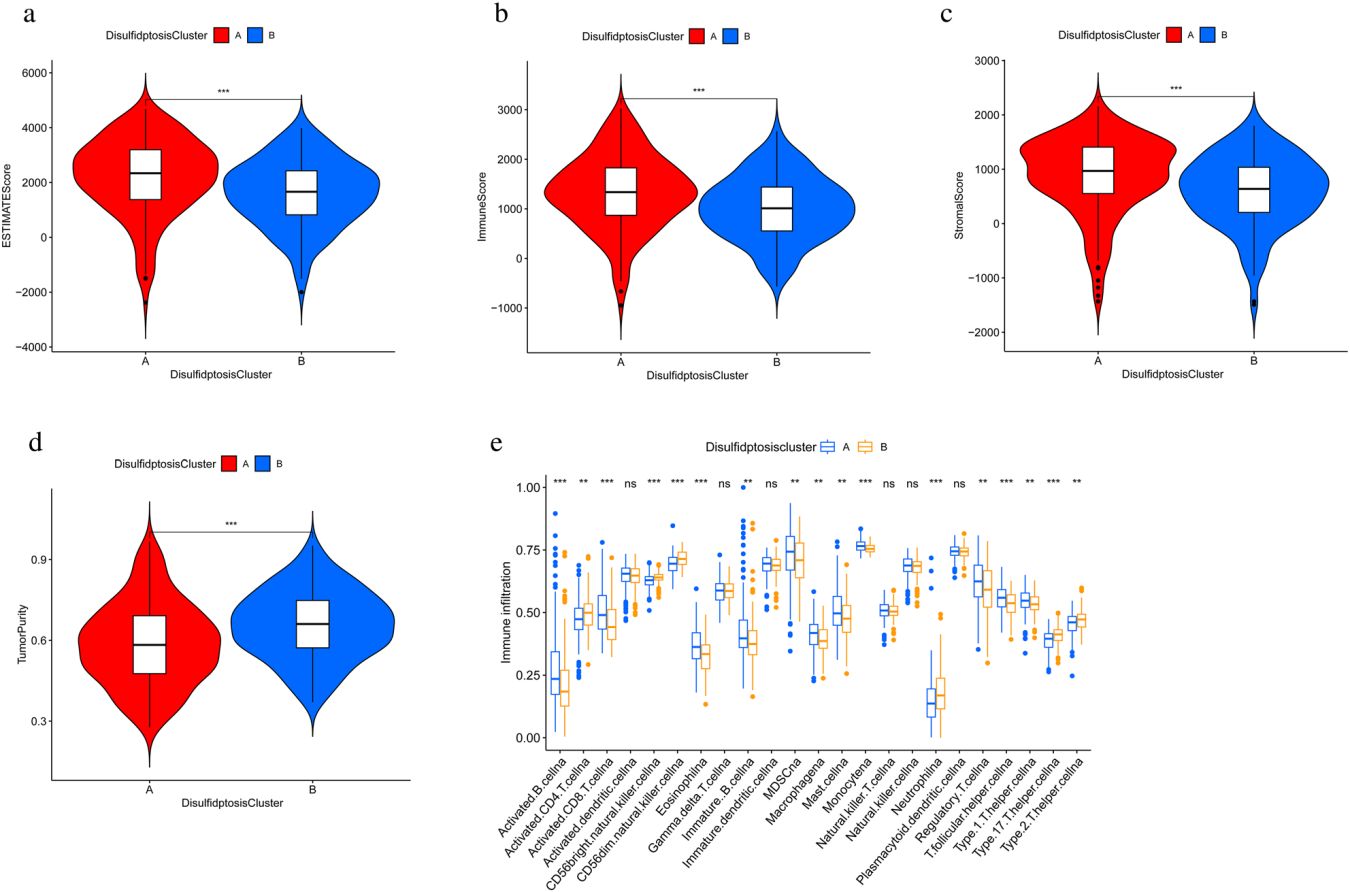
Analysis of TME in two subtypes (**a**–**d**) differences in estimates scores, immune scores, stormal scores and tumor purity by subtype. (**e**) differential distribution of 23 immune infiltrating cells in different subtypes of pancreatic cancer. *p < 0.05, **p < 0.01, ***p < 0.001.

Tumor development is closely related to the internal and external environment in which the tumor cells live. The TME generally consists of tumor cells, mesenchymal cells, and immune cells^14^. To characterize the two disulfidptosis subtypes in TME, we used the ESTIMATE algorithm to compare the differences in the degree of stromal cell and immune cell infiltration between the two subtypes. The results suggested that subtype A had higher immune cell scores, stromal cell scores, and estimates scores, while subtype B had higher tumor purity (Fig. 3a–d). In addition, we further assessed the differences in the enrichment of 23 immune infiltrating cells on different subtypes. The results were shown in Fig. 3e, where the majority of immune cells were more abundant in subtype A. All these results suggest that patients in subtype A are more immunocompetent.

### Identification of DEGs of the disulfidptosis subtypes and the development of a prognostic signature

**Figure 4:**
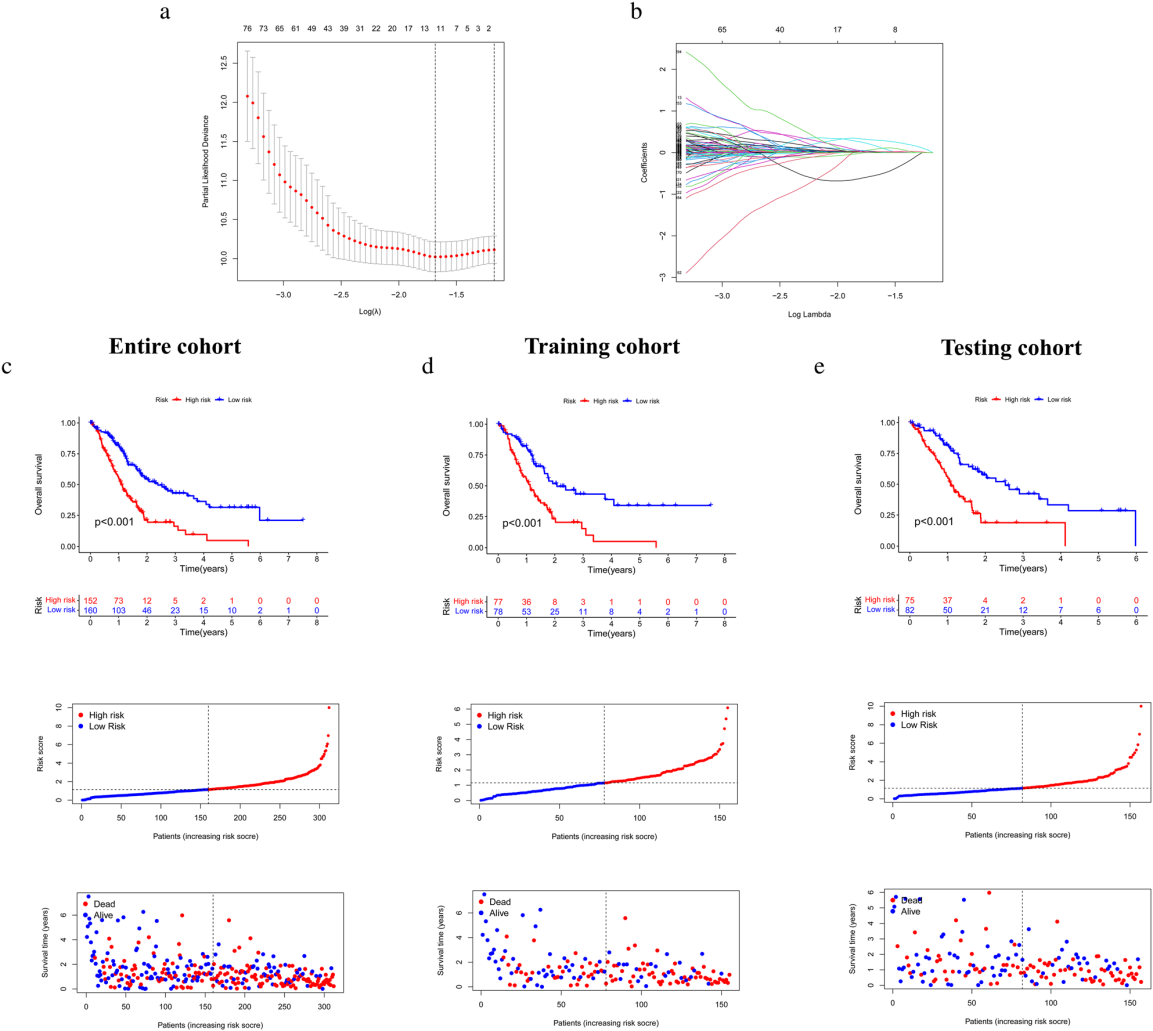
Construction of prognostic prediction signature (**a**,**b**) lasso regression results and cross-validation errors (**c**–**e**) Kaplan–Meier survival curve analysis, risk curve analysis, risk survival analysis for risk score groups in the entire, training and testing cohorts.

The disulfidptosis clustering typing is an excellent indicator of PCa's clinical outcome. A predictive signature was built to better characterize the hallmarks of disulfidptosis. Initially, we identified 3730 DEGs from the cluster by univariate COX analysis and identified 1416 DEGs associated with prognosis (Supplementary Table S3). We then randomized a total of 312 patients with clinical survival information 1:1 to obtain a training cohort (n = 155) and a testing cohort (n = 157). In the training cohort, Lasso-Cox regression analysis was performed on 1416 DEGs (Fig. 4a,b, Supplementary Table S4), resulting in the identification of five key genes: UCA1, FNDC3B, MYBL2, NHS, and CCDC15. We developed an RS formula for disulfidptosis based on these five genes. RS = UCA1 × 0.133 + FNDC3B × 0.835 + MYBL2 × 0.530 + NHS × 0.470 + CCDC15 × 0.505. We divided all PCa samples into HDR and LDR subgroups based on the median RS. The prognosis of the HDR group was noticeably poorer than that of the LDR group, as seen in Fig. 4d (*p* < 0.001). Risk curve analysis also showed that the risk of death in PCa patients increased as the RS increased. Risk survival analysis showed that patients with higher RS had shorter survival times. The above results were validated in both the entire cohort and the testing cohort (Fig. 4c and e).

**Figure 5:**
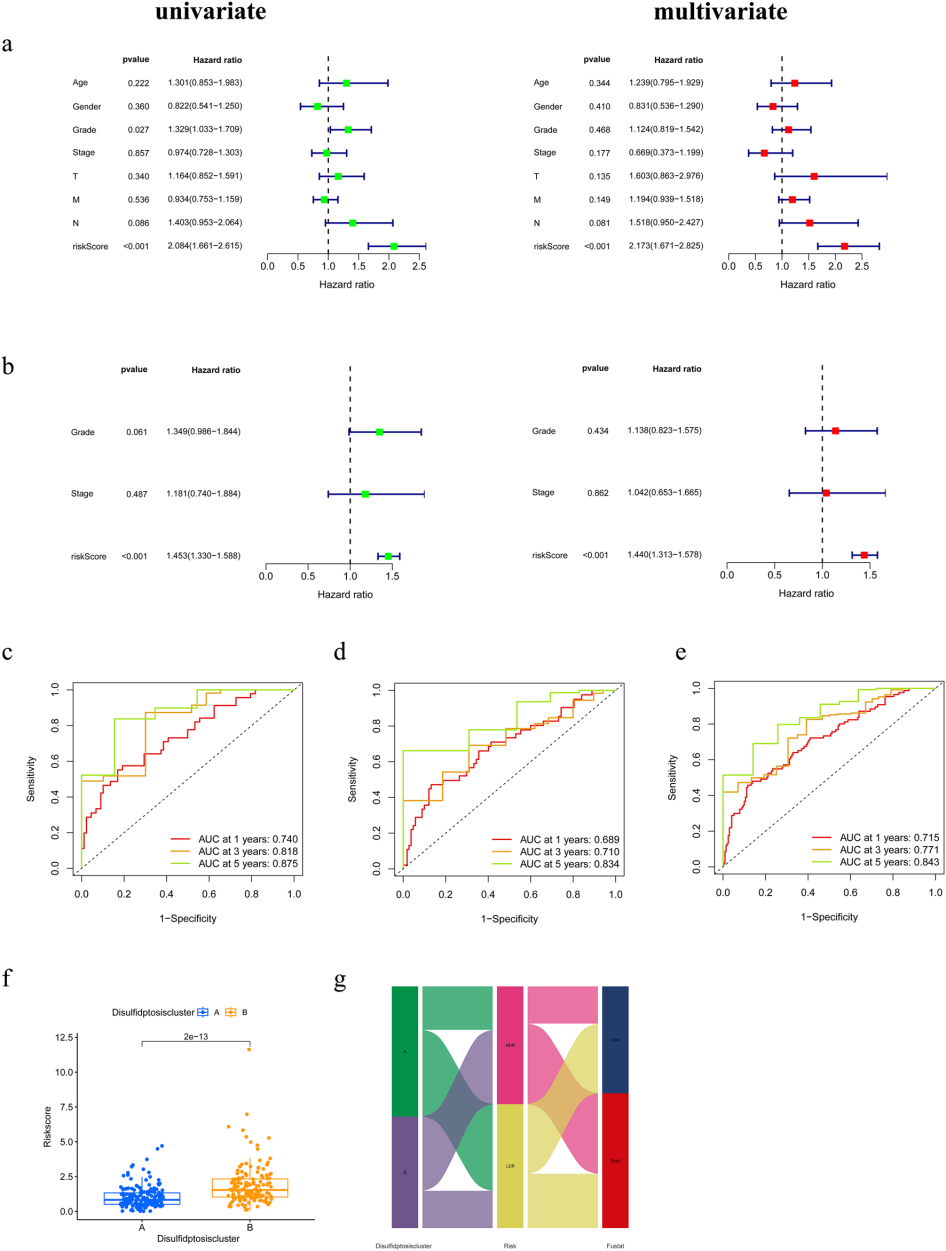
Validation of prognostic prediction signature (**a**) COX regression analysis based on TCGA-PAAD clinical traits. (**b**) COX regression analysis based on TCGA-PAAD and GSE183795 combined clinical traits. (**c**–**e**) 1, 3 and 5 years ROC curves for training cohort, testing cohort, and entire cohort. (**f**) analysis of differences in risk scores by typology. (**g**) Sankey diagram showing the relationship between subtypes, RS, and survival status.

To further confirm the validity of the risk signature, patients' prognostic risk scores and other relevant clinical characteristics were subjected to univariate and multifactorial Cox analyses. The results are shown in Fig. 5a,b, where the signature can be present as an independent risk factor both in the TCGA-PAAD-based clinical features alone and in the combined clinical features based on TCCA-PAAD and GSE183795 databases. Furthermore, we have confirmed the high accuracy of RS in predicting the prognosis of PCa patients at 1, 3, and 5 years. The AUC (area under the curve) for the training cohort was 0.740, 0.818, and 0.875 at 1, 3, and 5 years respectively (Fig. 5c). The AUC values for the internal testing cohort were 0.689 (1 year), 0.710 (3 years), and 0.834 (5 years) (Fig. 5d). The AUC values for the entire cohort at 1, 3, and 5 years were 0.715, 0.771, and 0.843 respectively (Fig. 5e). In addition, we found significantly lower RS in subtype A than in subtype B (Fig. 5f). And the Sankey plot shows (Fig. 5g) that patients in subtype B had higher RS and were more likely to die, which is consistent with previous analyses.

### Construction and verification of nomograms

**Figure 6:**
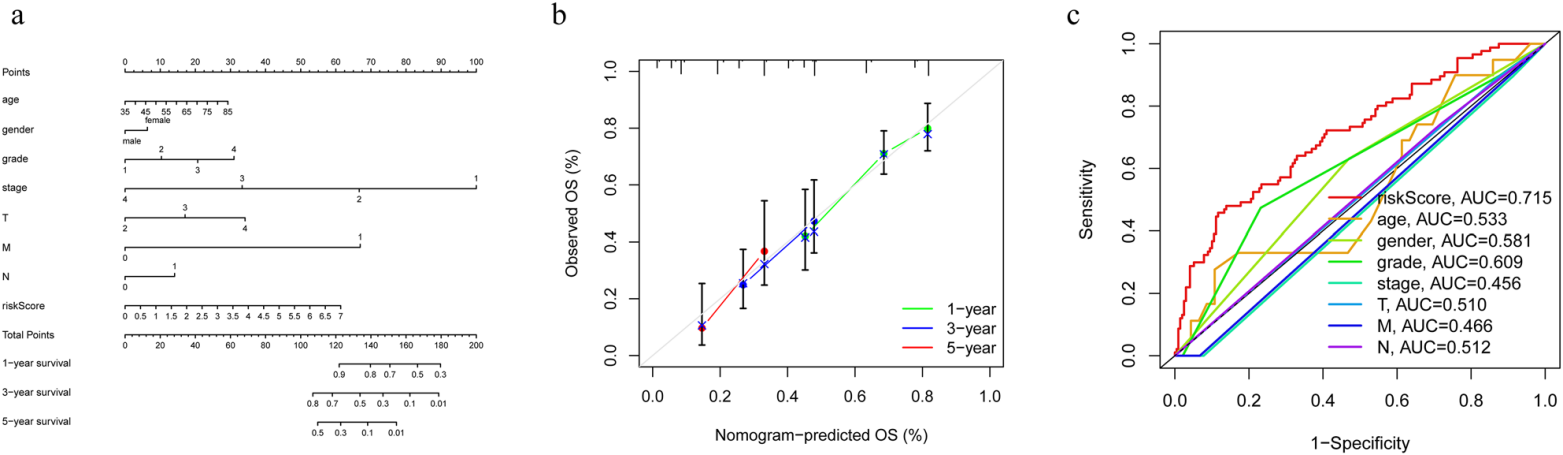
Nomogram and calibration curve. (**a**) Nomogram used to predict overall survival. (**b**) Calibration curves for 1-year, 3-year, and 5-year OS. (**c**) Area under the ROC curve for different clinical characteristics.

Considering clinical characteristics as indispensable factors in predicting patient prognosis^15^, we created a nomogram for predicting the incidence of 1, 3, and 5 OS based on factors such as RS and clinicopathology (Fig. 6a). And the calibration curve results showed good agreement between predicted OS and actual OS based on the nomogram (Fig. 6b). It further demonstrated the good clinical applicability of our developed signature. Not only that, we also plotted ROC curves for different clinicopathological features, and the results show that the AUC value of the risk score was the highest (Fig. 6c). These results further suggest that the accuracy of the risk model in predicting the prognosis of PCa patients is superior to other clinicopathological features.

### Functional enrichment analysis of disulfidptosis-related genes

**Figure 7:**
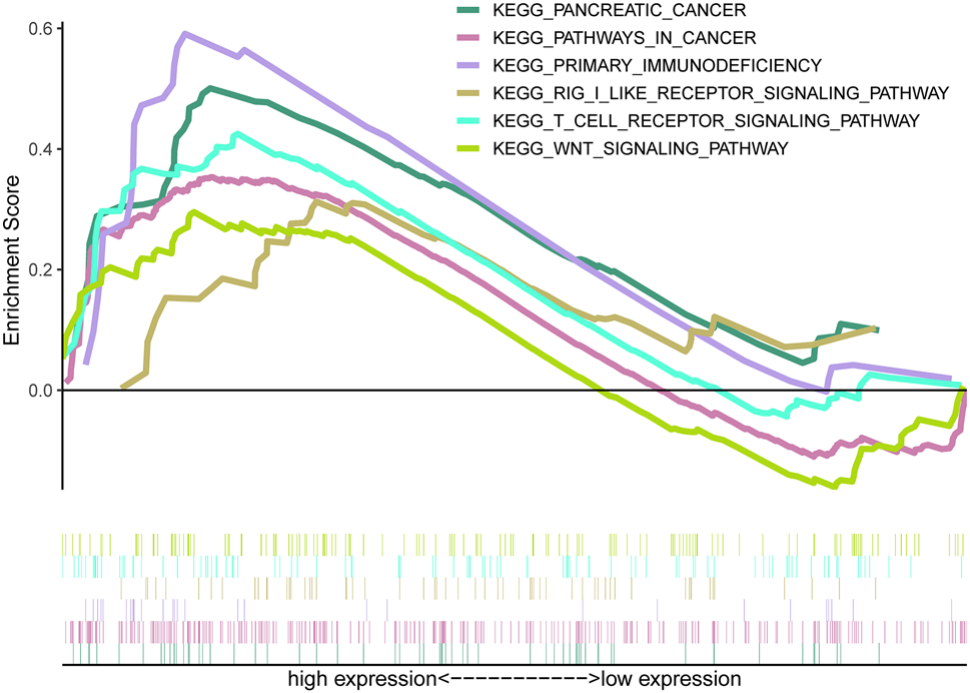
GSEA-KEGG enrichment analysis results.

To further understand the potential biological functions of disulfidptosis-related genes and their mechanisms, we performed GSEA-KEGG functional enrichment analysis of differential genes based on risk subgroups. The results are shown in Fig. 7 and were found to be mainly enriched in tumor-related and immune-related pathways: pancreatic cancer, pathways in cancer, primary immunodeficiency, rig i-like receptor (RLR) signaling pathway, t cell receptor signaling pathway, and Wnt signaling pathway. The RLR signaling pathway is a key signaling pathway in the intrinsic immune system for the clearance of RNA viruses and also plays an important role in tumor control by regulating NO concentration and metabolism^16^. The Wnt signaling pathway can mediate immunosuppression and promote the proliferation of PCa cells^17,18^. As a result, we attempted to perform an immune-related analysis based on the risk signature.

### Relationship between TME and immune status in different risk subgroups

**Figure 8:**
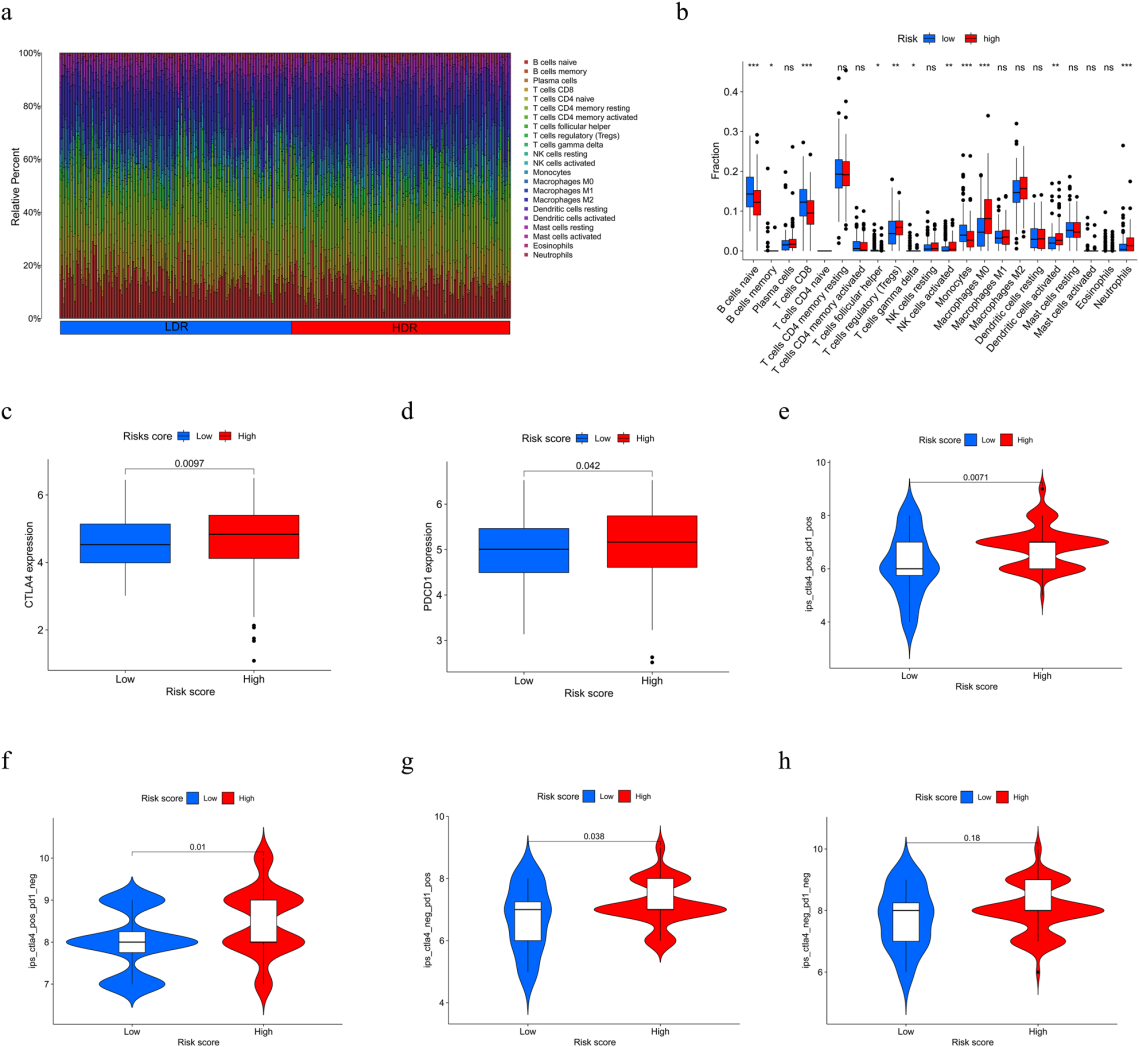
Correlation analysis between RS and TME (**a**,**b**) distribution and differential expression of different immune infiltrating cells in different risk subgroups. (**c**,**d**) differential analysis of the expression of immune checkpoint genes CTLA4 and PDCD1 in different risk subgroups. (**e**) CTLA4_positive + PD-1_positiv. (**f**) CTLA4_positive + PD-1_negative. (**g**) CTLA4_ negative + PD-1_positive. (**h**) CTLA4_negative + PD-1_negative. *p < 0.05, **p < 0.01, ***p < 0.001.

In this study, we used the CIBERSORT package to further analyze the differences between the different risk subgroups of PCa patients in terms of immune infiltrating cells. As shown in Fig. 8a,b, B cells naïve, B cells memory, T cells CD8, T cells follicular helper, T cells gamma delta, and monocytes were more abundant in the LDR group, while T cells regulatory, NK cells activated, Macrophages M0, Dendritic cells activated and Neutrophils were more abundant in the HDR group. These results suggest a strong link between TME and the prognosis of PCa patients. Immunotherapy for PCa has been the focus of attention, and immune checkpoints are important targets for immunotherapy. We further analyzed the expression of immune checkpoint genes CTLA4 and PDCD1 among different risk subgroups, as shown in Fig. 8c,d, CTLA4 and PDCD1 were more highly expressed in the HDR group. We then analyzed the efficacy of immunosuppressant treatment for these two immune checkpoints according to the immunophenotype scores of PCa patients obtained from the TCIA database and found that the HDR group would be better treated with either a PD1 inhibitor or a CTLA4 inhibitor or a combination of both (Fig. 8e–h).

### TMB and drug sensitivity analysis

**Figure 9:**
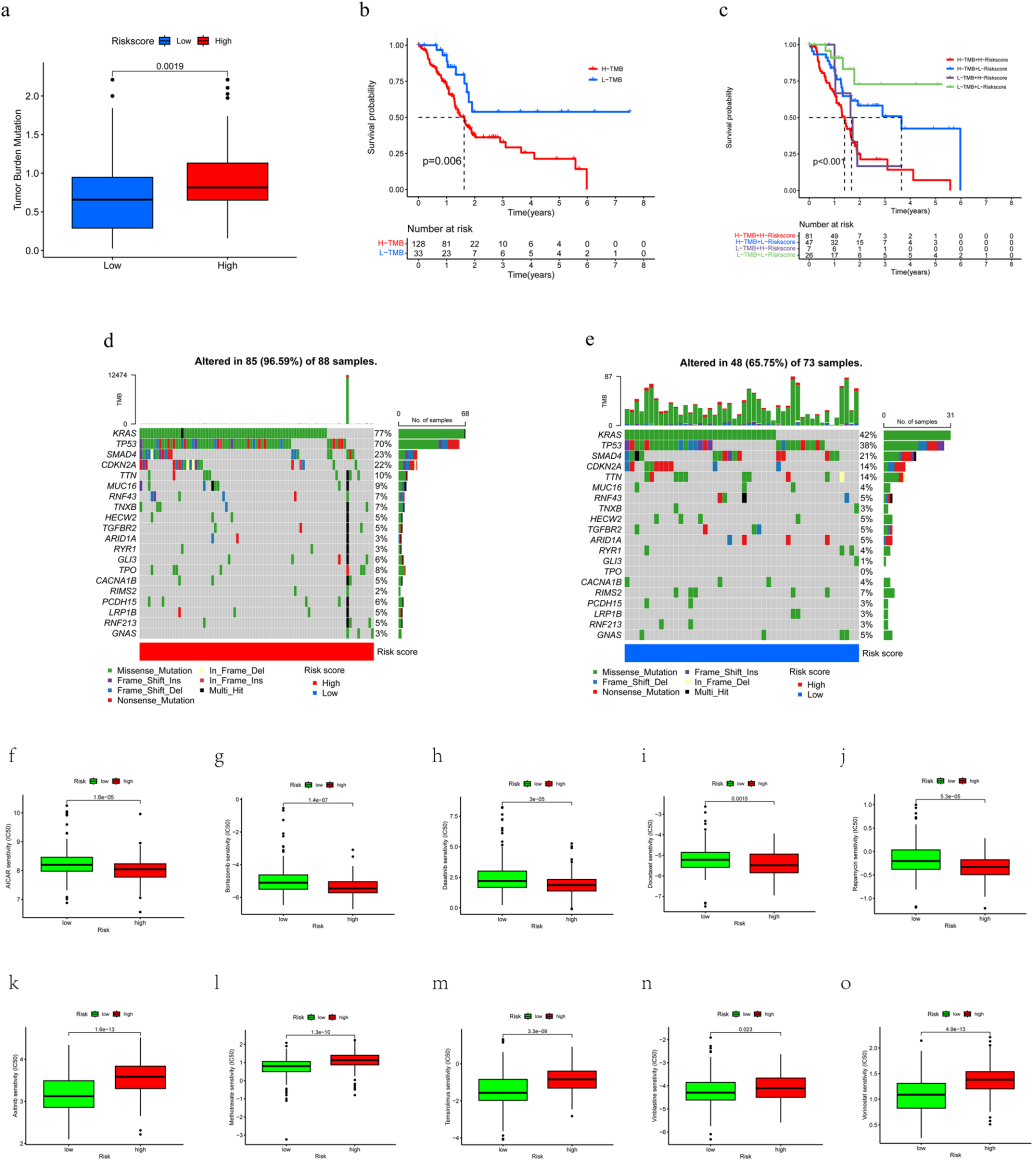
TMB and drug sensitivity analysis (**a**) TMB differences between different risk subgroups. (**b**) Kaplan–Meier survival curves between the high TMB and low TMB groups. (**c**) Kaplan–Meier survival curves between different groups. (**d**,**e**) Top 20 genes with the highest mutation frequency in the HDR and LDR subgroups. (**f**,**o**) The relationship between RS and drug sensitivity.

Immunotherapy has shown remarkable results in the clinical management of a wide range of malignancies, and TMB has shown great promise in predicting patient response to immunotherapy, with higher TMB often representing better immunotherapy outcomes^19,20^. We then further obtained data from TCGA-PAAD patients with mutational load (n = 161) and analyzed them. The results showed that the TMB was relatively higher in the TCGA-PAAD HDR group (Fig. 9a, *p* = 0.0019), which represents that the HDR group could have better immunotherapy outcomes. The Kaplan–Meier survival curves combining TMB and RS showed that PCa patients with higher TMB had a worse prognosis but were better treated with immunotherapy (Fig. 9b–c). The mutation profiles between the HDR and LDR subgroups were then compared. The mutation proportions were observed in up to 96.59% of the 88 HDR PCa patients (Fig. 9d) and 65.75% of the 73 LDR PCa patients (Fig. 9e), with KRAS, TP53, SMAD4, CKN2A, and TTN genes having high mutation proportions (> = 10%) in both risk subgroups.

Chemotherapy still plays an important role in PCa to date^21,22^, so we compared the IC50s of 138 standard chemotherapeutic agents from the 'pRRophetic' R package to determine the differences in sensitivity to these agents between HDR and LDR subgroups. All results are shown in Supplementary Table S5 and we further visualized five chemotherapeutic agents in each of the HDR and LDR subgroups that were commonly used and significantly different in PCa patients. The results suggest that five chemotherapeutic agents, AICAR, Bortezomib, Dasatinib, Docetaxel, and Rapamycin, may be more effective in treating PCa patients in the HDR group, while Axitinib, Methotrexate, Temsirolimus, Vinblastine, and Vorinostat are more effective in treating PCa patients in the LDR group (Fig. 9f–o).

### External validation, RT-qPCR and immunohistochemical analysis

**Figure 10:**
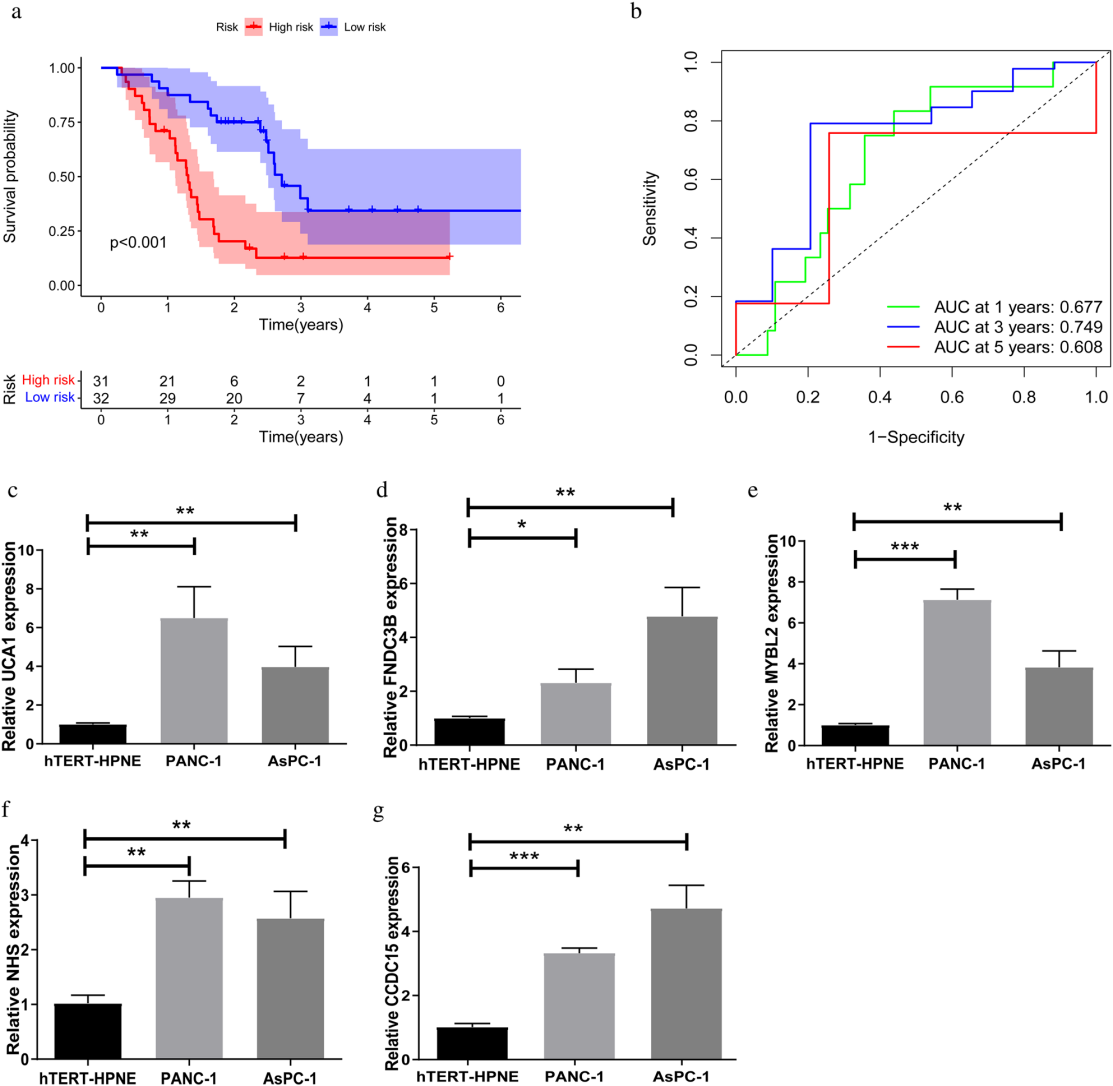
The results of external validation and RT-qPCR (**a**) Kaplan–Meier curves of OS in the HDR and the LDR subgroups in the GSE57495. (**b**) ROC analysis in the GSE57495 dataset. (**c**) UCA1. (**d**) FNDC3B. (**e**) MYBL2. (**f**) NHS. (**g**) CCDC15. *p < 0.05, **p < 0.01 and ***p < 0.001.

To further validate the role of five key genes (UCA1, FNDC3B, MYBL2, NHS, CCDC15) in the development of PCa in the risk model, the GSE57495 dataset was used as external validation. The Kaplan–Meier analysis showed that, as expected, patients in the HDR subgroup had significantly shorter OS than those in the LDR subgroup (*p* < 0.001) (Fig. 10a). The ROC curve results showed AUCs of 0.677, 0.749, 0.608 at 1, 3, and 5 years respectively (Fig. 10b). The expression levels of 5 risk grouping genes in hTERT-HPNE, PANC-1, and AsPC-1 cells were detected by RT-qPCR. The results are shown in Fig. 10c–g. The expression levels of all five genes were up-regulated in PCa cell lines. To better visualize the tissue expression of disulfidptosis-related genes, we extracted immunohistochemical maps of 12 typing genes and 5 risk grouping genes from the Human Protein Atlas website. The results are shown in Supplementary Figure S2.

## Discussion

As a highly malignant tumor, surgery is the main treatment for pancreatic cancer. However, due to the insidious onset of pancreatic cancer, lack of specific markers, and the susceptibility to early metastasis, patients with PCa often deprived of surgery once they are detected. At the same time, the postoperative recurrence rate is extremely high and the overall survival rate remains low^2,3^. With the development of immunotherapy in recent years, more and more patients with pancreatic cancer have new treatment options available to them^23^. Numerous researches have shown that the degree of immune cell infiltration has a strong correlation with the prognosis and treatment responsiveness of patients with PCa^24–26^. This then calls for further exploration of new therapeutic strategies, for which disulfidptosis is likely to be one of the research directions. Studies have shown that disulfidptosis can cause a series of redox defects and induce cell death and that the associated immune responses generated by immune cells are highly dependent on the occurrence of redox reactions^27,28^, and that the redox state is one of the important mechanisms of tumorigenesis and development^29^. Liu et al. similarly suggest that the induction of disulfidptosis may be an effective tumor treatment method^10^.

Extensive data show that CNV is strongly associated with cancer development^30^. Almost all cancers have copy number additions or deletions in certain regions of the chromosome. CNV of tumor-related genes can have a great influence on tumorigenesis and metastasis. For example, CNV on the GSTM1 gene lead to an increased risk of bladder cancer^31^. A copy number gains were also observed on chromosome 17q12-21, including the ERBB2 gene, a pathogenic CNV that drives gastric adenocarcinoma^32^. Pancreatic cancer patients harboring CNV of CDKN2A gene had significantly lower overall survival (OS) than pancreatic cancer patients not harboring CNV for this gene^33^. In this study, most disulfidptosis genes showed increased CNV mutation frequency and differential high expression in PCa, suggesting that CNV amplification frequency may promote overexpression of the gene. With the exception of FLNB and MYL6, the remaining 13 DRGs were significantly differentially expressed in the normal and tumor groups of the pancreas, and most DRGs shows higher expression in tumor groups. What we are considering is that in normal tissues, certain genes may play a tumor suppressor role, but in tumor tissues, the function of these genes may be altered to promote tumor development due to alterations in other genes or certain environmental factors^34^. For example, SLC7A11, a key gene for disulfidptosis, while promoting disulfidptosis in cancer tissues, is highly expressed in multiple tumors such as breast cancer, pancreatic cancer, and ovarian cancers, promotes cancer progression and metastasis. After further combining patient survival data, we identified 12 DRGs that are both differentially expressed and differentially survived in PCa. We clustered and typed these 12 DRGs by consensus clustering and found that they classified PCa patients into two disulfidptosis subtypes, A and B. These DRGs were more abundantly expressed in subtype B than in subtype A, and the prognosis was better in subtype A, suggesting that the DRGs are associated with poor patient prognosis. To further clarify the differences between the two disulfidptosis subtypes of immune infiltrating cells, we used the ESTIMATE algorithm to perform the analysis. The results found that subtype A had higher immune cell scores, stromal cell scores, and estimates scores, while subtype B had higher tumor purity. And most immune cells were more abundantly infiltrated in subtype A than in subtype B. Activated CD8+ T cells contribute to a durable and effective anti-tumor immune response^35^, and the greater abundance of CD8+ T cells in subtype A also means that subtype A patients are more immune competent and have a better prognosis.

To further explore the role of disulfidptosis signature in PCa, we obtained five key genes from DEGs between the two disulfidptosis subtypes by screening with Lasso–Cox regression analysis: UCA1, FNDC3B, MYBL2, NHS, CCDC15. Predictive risk signature based on these five key genes were constructed. Among them, UCA1 regulates the growth and metastasis of PCa through the adsorption of miR-135a^36^, and overexpression of MYBL2 may be associated with poor prognosis of PCa^37^. We divided PCa patients into different risk subgroups based on the median RS. ROC curves and COX regression analyses showed that the prediction signature had good predictive validity and could be used as an independent risk factor. The effectiveness of the prediction signature was further proved by internal and external validation. The prognosis of patients with PCa was then forecasted using a nomogram, and the graph was in good agreement with the predicted outcome. GSEA-KEGG analysis showed significant enrichment in pancreatic cancer pathways, cancer-related pathways, and immune-related pathways.

Based on these findings, we attempted an immune correlation analysis in the risk signature using the CiberSort algorithm. B cells, T cells, and monocytes were found to be more abundant in the LDR group, while regulatory T cells, activated NK cells, M0 macrophages, activated dendritic cells, and neutrophils were more abundant in the LDR group. An increased proportion of tumor-infiltrating regulatory T cells is associated with shorter survival in patients with PCa^38^, and TAMs (tumor-associated macrophages) can contribute to the angiogenesis and growth of pancreatic ductal adenocarcinoma^39^. The greater abundance of activated NK cells and activated dendritic cells in the HDR group may be due to the late use of immunotherapy and chemotherapy to stimulate the immune response in the HDR group^40,41^. It is suggested that these abnormally infiltrated immune cells may be associated with the development and poor prognosis of PCa. Checkpoint inhibitor immunotherapy, consisting of anti-CTLA4 and anti-PD1, is effective in the treatment of a variety of cancers^42–45^, and the feasibility of using immunotherapy to treat pancreatic cancer will receive increasing attention and research. Our study showed that CTLA4 and PDCD1 were more highly expressed in the HDR group. The results of the analysis of the efficacy of the immunosuppressive drugs used for these two immune checkpoints showed that the HDR group had better immunotherapy results. According to a research, individuals who received immunotherapy for tumors with elevated TMB had a better prognosis^20^, so we further analyzed the TMB of patients in different risk subgroups and found that patients with high-risk scores had more TMB. All of these findings imply that PCa patients in the HDR subgroup, despite having a poorer prognosis, may be better treated with immunotherapy, and it is recommended that patients in the HDR group be treated with immunotherapy as soon as possible. In addition, we further assessed the sensitivity of chemotherapeutic agents for PCa patients in the HDR and LDR subgroups and the results suggested that 50 of the 138 chemotherapeutic agents were not statistically different between the HDR and LDR subgroups and that 44 chemotherapeutic agents each were likely to be more effective for PCa patients in the HDR and LDR subgroups (Supplementary Table S5). Therefore, all the findings of this study can be used to tailor the administration of chemotherapeutic drugs to PCa patients in various risk subgroups, improving patient prognosis.

However, this study still has some shortcomings and limitations. Firstly, although this study has been validated using several independent cohorts, more independent cohorts and larger sample sizes are needed for subsequent validation. Secondly, we did not further investigate this gene set in other cancer types due to limited conditions. Also, the clustering algorithm used in this study is relatively homogeneous, and subsequent iterative validation of more personalized grouping algorithms, such as multidimensional scaling (MDS)-based clustering^46^, is still needed to enhance its practical applicability. Finally, as a retrospective study, more prospective studies and basic studies are needed to support and validate the phase results of this study.

## Conclusion

In this study, we applied the subtyping model of disulfidptosis to pancreatic cancer for the first time and constructed a risk signature based on the disulfidptosis subtype, which can provide an accurate assessment of prognosis, immunotherapy, and chemotherapy response in PCa patients, opening up new targets and directions for the prognosis and treatment of pancreatic cancer.

### Supplementary Information

Supplementary Figure S1.

Supplementary Figure S2.

Supplementary Table S1.

Supplementary Table S2.

Supplementary Table S3.

Supplementary Table S4.

Supplementary Table S5.

## Data Availability

The data used to support the findings of this study are available from the corresponding author upon request.
