# Supplementary material for: Bioinformatics-based analysis of the relationship between disulfidptosis and prognosis and treatment response in pancreatic cancer
Source: Sci Rep. 2023 Dec 14;13:22218. doi: 10.1038/s41598-023-49752-4 (PMC10721597; doi:10.1038/s41598-023-49752-4)
Supplement: Supplementary file 1 — Supplementary Figure S1. [file 41598_2023_49752_MOESM1_ESM.docx]

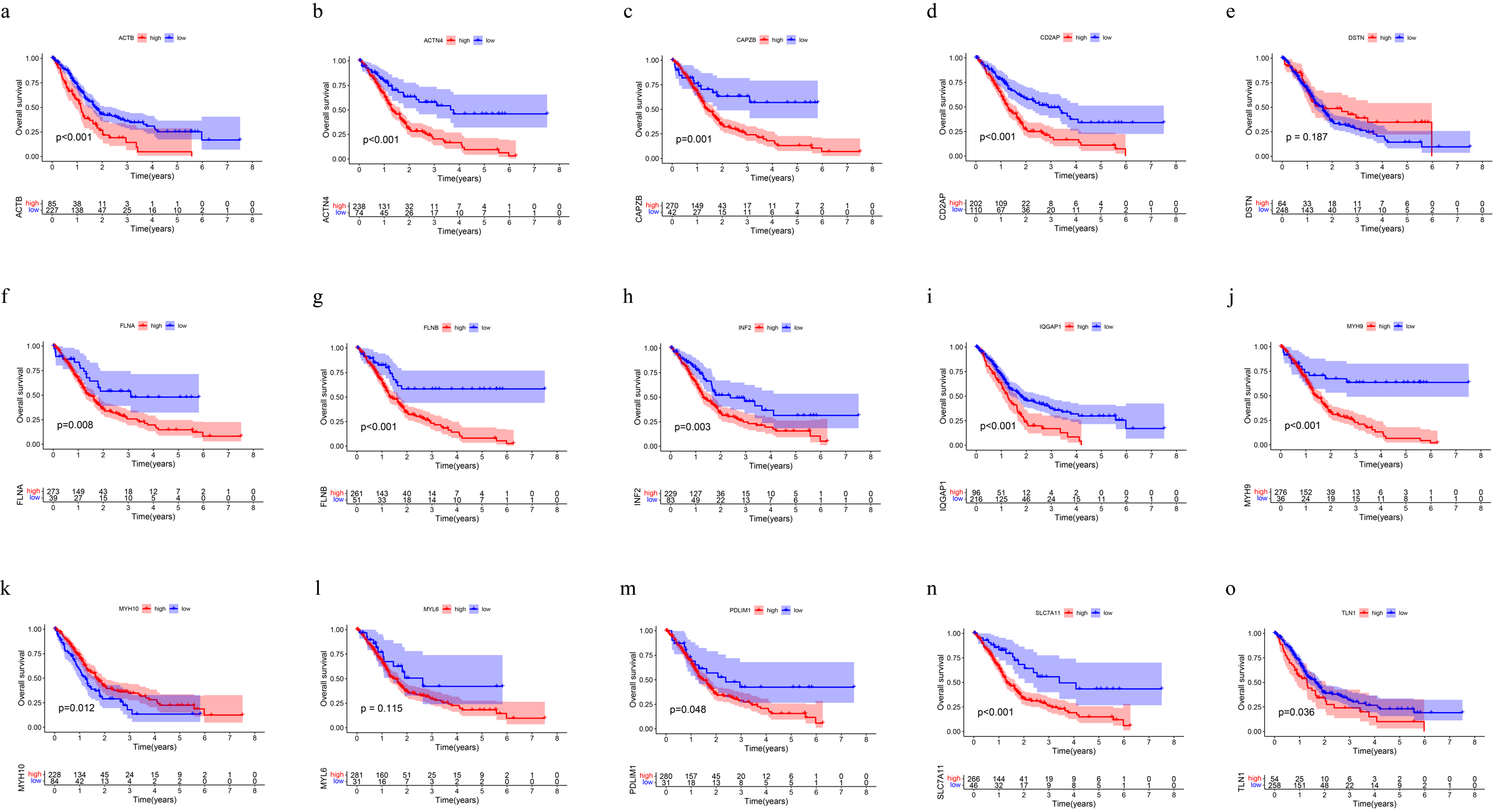


Supplementary Figure S1 Kaplan-Meier survival curve analysis results for 15 DRGs. (a) ACTB. (b) ACTN4. (c) CAPZB. (d) CD2AP. (e) DSTN. (f) FLNA. (g) FLNB. (h) INF2. (i) IQGAP1. (j) MYH9. (k) MYH10. (l) MYL6. (m) PDLIM1. (n) SLC7A11. (o) TLN1.
