# Supplementary material for: Bioinformatics-based analysis of the relationship between disulfidptosis and prognosis and treatment response in pancreatic cancer
Source: Sci Rep. 2023 Dec 14;13:22218. doi: 10.1038/s41598-023-49752-4 (PMC10721597; doi:10.1038/s41598-023-49752-4)
Supplement: Supplementary file 2 — Supplementary Figure S2. [file 41598_2023_49752_MOESM2_ESM.docx]

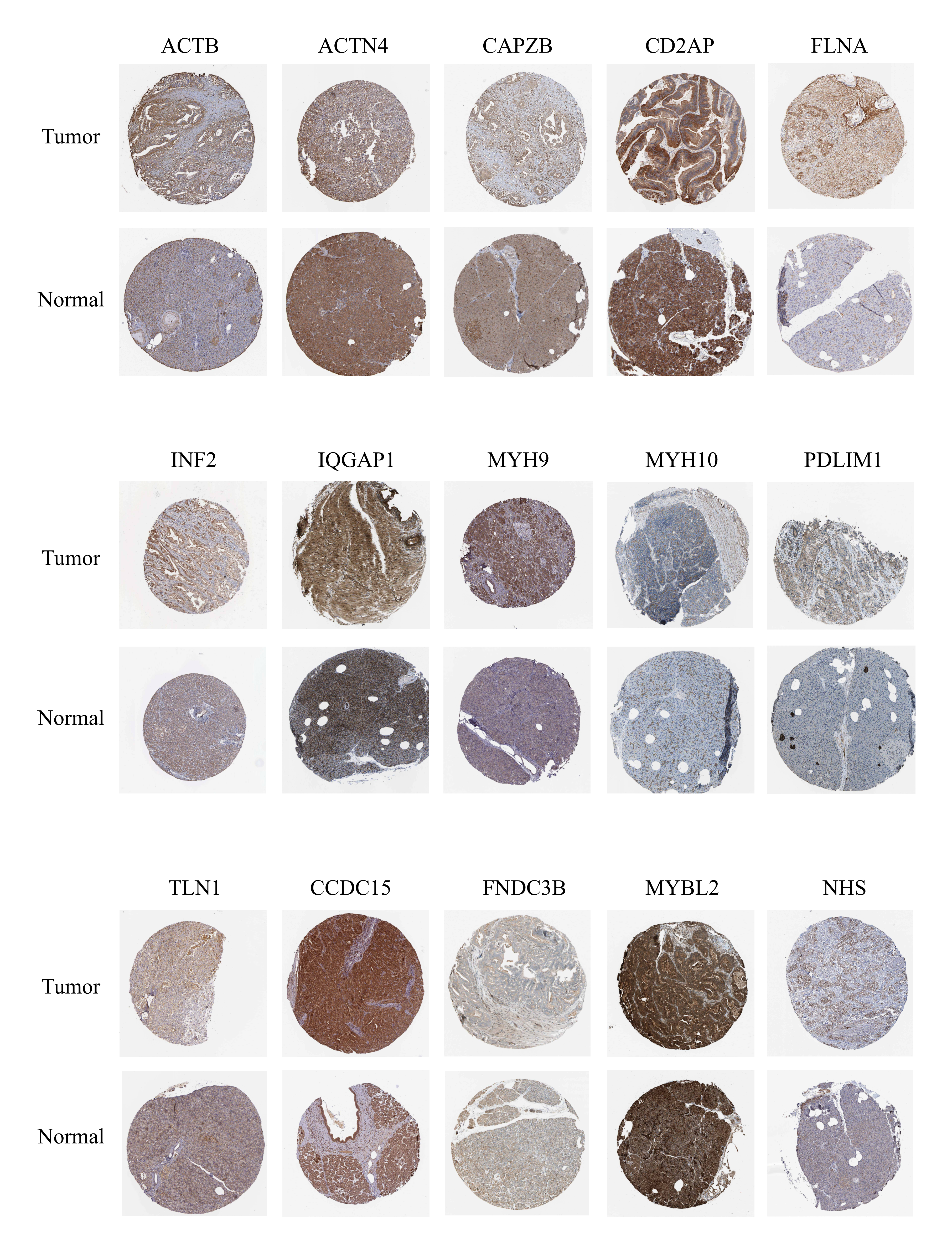


Supplementary Figure S2 Extraction of immunohistochemical profiles from the Human Protein Atlas website (SLC7A11 and UCA1 were not available from the website).
