# Supplementary material for: Bioinformatics-based analysis of the relationship between disulfidptosis and prognosis and treatment response in pancreatic cancer
Source: Sci Rep. 2023 Dec 14;13:22218. doi: 10.1038/s41598-023-49752-4 (PMC10721597; doi:10.1038/s41598-023-49752-4)
Supplement: Supplementary file 3 — Supplementary Table S1. [file 41598_2023_49752_MOESM3_ESM.docx]

Supplementary Table S1. Demographics and related clinical information of pancreatic cancer patients in different databases

| Character | | TCGA | GTEx | GSE183795 | GSE57495 |
| --- | --- | --- | --- | --- | --- |
|  |  | n=178 | n=167 | n=134 | n=63 |
| Age |  | | none | none | none |
| <=65 | 94(52.81%) | |  |  |  |
| >65 | 84(47.19%) | |  |  |  |
| Gender |  | | none | none | none |
| Female | 80(44.94%) | |  |  |  |
| Male | 98(55.06%) | |  |  |  |
| Grade |  | | none |  | none |
| G1 | 31(17.42%) | |  | 3(2.24%) |  |
| G2 | 95(53.37%) | |  | 75(55.97%) |  |
| G3 | 48(26.97%) | |  | 53(39.55%) |  |
| G4 | 2(1.12%) | |  | 2(1.49%) |  |
| unkonwn | 2(1.12%) | |  | 1(0.75%) |  |
| Stage |  | | none |  | none |
| Stage I | 21(11.8%) | |  | 8(5.97%) |  |
| Stage II | 147(82.58%) | |  | 103(76.86%) |  |
| Stage III | 3(1.69%) | |  | 16(11.94%) |  |
| Stage IV | 4(2.25%) | |  | 6(4.48%) |  |
| unknown | 3(1.69%) | |  | 1(0.75%) |  |
| T |  | | none | none | none |
| T1 | 7(3.93%) | |  |  |  |
| T2 | 24(13.48%) | |  |  |  |
| T3 | 142(79.78%) | |  |  |  |
| T4 | 3(1.69%) | |  |  |  |
| unknown | 2(1.12%) | |  |  |  |
| M |  | | none | none | none |
| M0 | 80(44.94%) | |  |  |  |
| M1 | 4(2.25%) | |  |  |  |
| unknown | 94(52.81%) | |  |  |  |
| N |  | | none | none | none |
| N0 | 49(27.53%) | |  |  |  |
| N1 | 124(69.66%) | |  |  |  |
| unknown | 5(2.81%) | |  |  |  |
