# Supplementary material for: Bioinformatics-based analysis of the relationship between disulfidptosis and prognosis and treatment response in pancreatic cancer
Source: Sci Rep. 2023 Dec 14;13:22218. doi: 10.1038/s41598-023-49752-4 (PMC10721597; doi:10.1038/s41598-023-49752-4)
Supplement: Supplementary file 4 — Supplementary Table S2. [file 41598_2023_49752_MOESM4_ESM.docx]

**Supplementary Table S2 Primer sequences**

| Gene id | Primer F | Primer R | |
| --- | --- | --- | --- |
| UCA1 | 5′-CCGAGAGCCGATCAGACAAA-3′ | 5′-TGGGATGGCCATTTGGAAGG-3′ | |
| FNDC3B | 5′-ATAGCCAAGAGGTGGTGTGC-3′ | 5′-TACTCCACTGCAACGTGACC-3′ |  |
| MYBL2 | 5′-CATGAGGAGAACCGCACTGA-3′ | 5′-TCAGGGTTGAGGTGGTTGTG-3′ |  |
| NHS | 5′-CTGCTGCATGCCCAAGAATG-3′ | 5′-GCGCGGTAGTACACACTCAG-3′ |  |
| CCDC15 | 5′-TTCCAAGCTCCACTGGCATT-3′ | 5′-TCCTCTGCTCTGCAGCATAA-3′ |  |
