# Supplementary material for: Bioinformatics-based analysis of the relationship between disulfidptosis and prognosis and treatment response in pancreatic cancer
Source: Sci Rep. 2023 Dec 14;13:22218. doi: 10.1038/s41598-023-49752-4 (PMC10721597; doi:10.1038/s41598-023-49752-4)
Supplement: Supplementary file 5 — Supplementary Table S3. [file 41598_2023_49752_MOESM5_ESM.docx]

**Supplementary Table S3 1416 DEGs associated with prognosis**

| A1BG | A2ML1 | ABCA10 | ABCA5 | ABCC1 | ABCC3 | ABCC8 | ABHD14A | ABHD15 | ABTB2 |  |
| --- | --- | --- | --- | --- | --- | --- | --- | --- | --- | --- |
| ACADVL | ACSL5 | ACSL6 | ACTBL2 | ACTL6A | ACTL6B | ACTN4 | ACTR3 | ADAM10 | ADAM9 | |
| ADAMTSL2 | ADAMTSL5 | ADM | ADORA2A | ADORA2B | AFAP1 | AGER | AGFG1 | AGMO | AGPS | |
| AGRN | AHNAK2 | AHR | AK4 | ALDH3B1 | ALDH9A1 | ALG3 | ALKBH5 | ALPK1 | AMBRA1 | |
| AMZ2 | AMZ2P1 | ANAPC1 | ANAPC16 | ANAPC2 | ANGEL1 | ANKRD16 | ANKRD22 | ANKRD27 | ANKRD50 | |
| ANLN | ANXA11 | ANXA2 | ANXA2P1 | ANXA2P2 | ANXA2P3 | ANXA3 | AP1S3 | AP3B2 | AP3S1 | |
| APLP1 | APLP2 | APOBEC1 | APOL1 | APPBP2 | AQP5 | ARAP2 | AREG | ARHGAP11A | ARHGAP21 | |
| ARHGAP26 | ARHGAP32 | ARL14 | ARL6IP6 | ARMC10 | ARMC12 | ARMC2 | ARNT2 | ARNTL2 | ARPC2 | |
| ASAP2 | ASB16 | ASF1B | ASPH | ASPM | ASTN1 | ATAD2 | ATCAY | ATG16L1 | ATIC | |
| ATP2A3 | ATP6V0E2-AS1 | ATP6V1G2 | ATP8A1 | ATR | ATRNL1 | AUNIP | AURKA | AURKB | B2M | |
| B3GNT2 | B3GNT5 | B3GNT7 | B4GALT5 | BAK1 | BARD1 | BAZ1A | BCL10 | BCL2L1 | BCL2L14 | |
| BCL2L15 | BCL9L | BDH1 | BEAN1 | BEND5 | BEX4 | BFAR | BHLHE40 | BIK | BIRC5 | |
| BLM | BMP2 | BMP4 | BORA | BRCA1 | BRCA2 | BRCC3 | BRIP1 | BSN | BTBD16 | |
| BTBD6 | BUB1 | BUB1B | BZW1 | BZW2 | C12orf56 | C12orf57 | C16orf87 | C17orf53 | C19orf33 | |
| C1orf112 | C1QTNF4 | C22orf39 | C22orf42 | C4BPB | C6orf132 | C6orf223 | C7orf26 | C8orf74 | CA11 | |
| CA12 | CAB39 | CABP4 | CACNA1A | CACNA1B | CACNA2D2 | CALY | CAMK2B | CAMTA2 | CAP1 | |
| CAPG | CAPN1 | CAPN2 | CAPRIN1 | CARD11 | CARS | CASD1 | CASK | CASKIN2 | CASP10 | |
| CASP2 | CATSPER1 | CAV2 | CBX3 | CBX7 | CCDC106 | CCDC15 | CCDC158 | CCDC68 | CCDC80 | |
| CCL20 | CCNA2 | CCNB1 | CCNB2 | CCND1 | CCNE1 | CCNF | CD109 | CD151 | CD2AP | |
| CD58 | CD9 | CDA | CDC20 | CDC25C | CDC45 | CDC6 | CDC73 | CDCA2 | CDCA4 | |
| CDCA5 | CDCA8 | CDCP1 | CDH10 | CDH3 | CDK1 | CDK12 | CDK2 | CDK6 | CDK9 | |
| CDKN3 | CDO1 | CDR2L | CEACAM6 | CELF3 | CELF4 | CELSR1 | CENPA | CENPE | CENPF | |
| CENPI | CENPK | CENPN | CEP152 | CEP55 | CERK | CES4A | CFL1 | CHD5 | CHEK1 | |
| CHML | CHMP2B | CHRNB2 | CIRBP | CIT | CKAP2L | CKAP5 | CKS1B | CKS2 | CLASP2 | |
| CLDN1 | CLDN4 | CLEC4F | CLIC1 | CLIC3 | CLIP3 | CLSPN | CNIH2 | CNKSR2 | CNN3 | |
| CNNM4 | CNOT1 | CNOT6 | CNPY3 | COL17A1 | COPRS | COPS3 | COQ10A | CORO2A | COX6B2 | |
| CPE | CRCT1 | CREBL2 | CREG2 | CRHBP | CRIP3 | CRMP1 | CRTC1 | CRY2 | CRYBA2 | |
| CSAD | CSNK2A3 | CSTF2 | CTC1 | CTNNA1 | CTNND1 | CTTN | CTTNBP2NL | CXorf38 | CYB5B | |
| CYB5D2 | CYFIP2 | CYP2S1 | CYP46A1 | CYP4F11 | DAAM2 | DCAKD | DCT | DCTN5 | DCX | |
| DDIT4 | DDX18 | DDX24 | DDX25 | DDX5 | DDX60 | DDX60L | DEF8 | DENND4B | DEPDC1 | |
| DEPDC1B | DERA | DGCR6L | DHFR | DHRS9 | DIAPH1 | DIAPH3 | DIRAS1 | DKK1 | DLAT | |
| DLG5 | DLGAP3 | DLGAP5 | DMAP1 | DNAH3 | DNAJC18 | DNAJC6 | DNMT3A | DOCK5 | DOCK9 | |
| DPH5 | DPM3 | DPP3 | DPP7 | DPY19L1 | DPY19L2P4 | DSC2 | DSCAM | DSCC1 | DSG2 | |
| DSG3 | DTL | DTX3 | DTX3L | DTX4 | DUSP26 | DVL2 | DYNC1I1 | DZIP3 | E2F1 | |
| E2F7 | E2F8 | ECM1 | ECT2 | EFNA4 | EFNA5 | EFNB1 | EFNB2 | EFR3B | EIF2S2P4 | |
| EIF5A2 | ELAC1 | ELAVL4 | ELF4 | ELOVL1 | ELOVL4 | ELP5 | EME1 | EML6 | ENDOD1 | |
| ENO1 | EPHA2 | EPHB4 | EPHX4 | EPS8 | EPS8L1 | ERBB2 | ERCC6L | EREG | ERI1 | |
| ERLIN1 | ERP29 | ERVMER34-1 | ESCO2 | ESPL1 | ESYT2 | ETV6 | ETV7 | EVPL | EXO1 | |
| EXT1 | EZH2 | EZR | F10 | F13A1 | F2RL1 | F3 | FADD | FAM104B | FAM110B | |
| FAM111B | FAM114A1 | FAM117A | FAM129B | FAM135B | FAM160A1 | FAM172A | FAM19A2 | FAM19A4 | FAM210A | |
| FAM222A | FAM53B | FAM72B | FAM83A-AS1 | FAM83B | FAM83D | FANCB | FANCD2 | FBF1 | FBLL1 | |
| FBRSL1 | FBXO34 | FBXO9 | FBXW4 | FEN1 | FER1L4 | FERMT1 | FGD6 | FGF10 | FGF14 | |
| FGFBP1 | FHL2 | FIG4 | FLNB | FLRT3 | FLYWCH2 | FMN1 | FMN2 | FNDC3B | FOXL1 | |
| FOXM1 | FOXQ1 | FRMD5 | FRRS1 | FRRS1L | FTSJ1 | FUNDC2 | FUT8 | FXR2 | FXYD6 | |
| FZD6 | GABARAP | GABARAPL2 | GABRG2 | GABRP | GALNT16 | GALNT5 | GAN | GART | GATAD2A | |
| GBP3 | GDAP1 | GDE1 | GDF11 | GGA2 | GID8 | GINS1 | GJB2 | GJB4 | GJB5 | |
| GJB6 | GKAP1 | GLCCI1 | GLRX3 | GMPS | GNA15 | GNAZ | GNG4 | GNG7 | GOLGA8B | |
| GORASP2 | GPD2 | GPLD1 | GPN1 | GPR162 | GPR39 | GPR75 | GPR87 | GPRASP1 | GPRASP2 | |
| GPRC5A | GPS2 | GPSM2 | GPX3 | GREB1L | GRHL2 | GRIA2 | GSDMC | GSK3B | GSTA4 | |
| GSTP1 | GTF2IRD1 | GTPBP2 | GTSE1 | GULP1 | GYG1 | H1F0 | HCAR1 | HCAR3 | HDAC5 | |
| HDDC2 | HDGF | HDHD2 | HEATR1 | HFM1 | HHIP | HHLA2 | HILPDA | HIST1H1B | HIST1H1C | |
| HIST1H1D | HIST1H2AG | HIST1H2AI | HIST1H2AL | HIST1H2BC | HIST1H2BE | HIST1H2BK | HIST1H2BO | HIST1H3C | HIST1H3H | |
| HIST1H4H | HIST1H4I | HJURP | HK1 | HK2 | HKDC1 | HLA-B | HLA-C | HLF | HMGA1 | |
| HMGA1P1 | HMGA2 | HMGCLL1 | HMGCS1 | HMGN3 | HMGXB4 | HMMR | HNRNPF | HOXB5 | HOXB6 | |
| HOXB7 | HPCAL1 | HPDL | HRASLS2 | HRH1 | HS3ST1 | HSD17B14 | HTATIP2 | HTR1D | IFI27 | |
| IFITM10 | IGF2BP2 | IGF2BP3 | IGIP | IGSF3 | IGSF9 | IL11RA | IL17RE | IL18 | IL1A | |
| IL1RAP | IL1RN | IL20RB | INA | INCENP | ING5 | INPP4B | INPP5K | INSIG2 | INSM1 | |
| IQGAP1 | IQGAP3 | IQSEC3 | IRAK2 | IRAK4 | IRS1 | IRX5 | ISCA1 | ISCU | ITCH | |
| ITGA2 | ITGA3 | ITGA6 | ITGB4 | ITGB6 | ITPR3 | ITPRIPL2 | JPH3 | JRKL | JUN | |
| JUP | KATNAL2 | KATNBL1 | KCMF1 | KCNB2 | KCNC3 | KCNH3 | KCNIP3 | KCNJ11 | KCNK6 | |
| KCNMB2 | KCNN4 | KCTD2 | KCTD5 | KDM5B | KIAA1522 | KIAA1586 | KIF11 | KIF13B | KIF14 | |
| KIF15 | KIF18A | KIF18B | KIF20A | KIF20B | KIF23 | KIF2C | KIF4A | KIF4B | KIF5A | |
| KIF5C | KLF3 | KLF5 | KLHDC1 | KLHL22 | KLK11 | KLK6 | KLK7 | KLK8 | KNOP1 | |
| KNSTRN | KNTC1 | KPNA5 | KPNA7 | KRT13 | KRT15 | KRT16 | KRT17 | KRT18 | KRT18P28 | |
| KRT19 | KRT7 | LACTB | LACTB2 | LAD1 | LAMA3 | LAMB3 | LAMC2 | LANCL3 | LASP1 | |
| LCMT2 | LDHA | LEMD1 | LGALS3 | LIN9 | LINC00158 | LINC00266-1 | LINC00346 | LINC00476 | LIPH | |
| LIPM | LMNB1 | LMNB2 | LMO7 | LONRF2 | LOXL2 | LPAR5 | LPCAT2 | LRCH1 | LRP10 | |
| LRRC4B | LRRC57 | LRRC8A | LRRC8E | LSM12 | LUC7L | LY6E | LYRM1 | LYRM9 | LYSMD2 | |
| MACC1 | MAD2L1 | MALL | MAP1LC3A | MAP2 | MAP3K13 | MAP3K15 | MAP4K4 | MAP6 | MAPK4 | |
| MAPK8IP2 | MAST3 | MASTL | MB | MBD3 | MBOAT2 | MCM10 | MCM2 | MCM4 | MCM8 | |
| MCOLN1 | MCU | MECOM | MELK | MET | METAP1 | METTL16 | METTL7A | MGAT4B | MGLL | |
| MGST1 | MICAL2 | MIF4GD | MIR600HG | MIR7-3HG | MKI67 | MLKL | MMEL1 | MMP1 | MMP12 | |
| MMP13 | MMP14 | MOAP1 | MOCOS | MOCS2 | MOGAT3 | MOSPD1 | MPP2 | MPZL2 | MPZL3 | |
| MRAS | MROH8 | MRPL15 | MRPS10 | MSH2 | MSI1 | MSLN | MST1R | MSX2 | MSX2P1 | |
| MT1M | MTDH | MTFR1 | MTFR2 | MTHFD1 | MTHFD2 | MTMR2 | MTMR7 | MUC16 | MUC21 | |
| MUC4 | MVP | MXD1 | MYBL2 | MYD88 | MYEF2 | MYEOV | MYH16 | MYL12A | MYL12B | |
| MYL6B | MYO1E | MYO5B | MYOF | NAP1L1 | NAP1L2 | NAP1L3 | NAP1L5 | NCALD | NCAPD3 | |
| NCAPG | NCAPG2 | NCAPH | NCDN | NCEH1 | NCK1 | NCOA5 | NDC1 | NDC80 | NDE1 | |
| NDUFB8 | NDUFV3 | NEFM | NEIL3 | NEK2 | NEK7 | NENF | NET1 | NEURL4 | NFE2L3 | |
| NGEF | NHS | NIP7 | NIPAL1 | NIPSNAP3B | NISCH | NKIRAS1 | NLGN1 | NLN | NMI | |
| NMU | NOL4 | NOVA1 | NOVA2 | NPAS2 | NQO1 | NRIP2 | NT5C3B | NTF4 | NUAK2 | |
| NUDT9P1 | NUF2 | NUP93 | NUSAP1 | NXF1 | OAS1 | OAS3 | OASL | OIP5 | OLA1 | |
| OMP | OPLAH | OR7E91P | ORC1 | ORC6 | ORMDL1 | OSBPL10 | OSGIN2 | OVOL1 | OXSR1 | |
| OXTR | P2RX1 | P2RY2 | P4HA1 | PADI1 | PAFAH1B1 | PAFAH1B2 | PAIP2 | PAK3 | PALB2 | |
| PAM | PAQR4 | PARP4 | PARPBP | PATL1 | PAWR | PBK | PBXIP1 | PCDH1 | PCDH19 | |
| PCDH7 | PCF11 | PCSK1N | PDCD10 | PDCD6IP | PDIA4 | PDK1 | PDZD4 | PDZK1IP1 | PEBP1 | |
| PEG3 | PEMT | PERP | PEX13 | PFDN5 | PGAM1 | PGK1 | PGM2 | PGM2L1 | PHF10 | |
| PHF21B | PHF23 | PHF6 | PHLDA1 | PHYHD1 | PI3 | PI4KB | PIEZO1 | PIGA | PIK3CB | |
| PIPOX | PITPNA | PKD1L2 | PKM | PKMYT1 | PKP3 | PLA2G16 | PLA2G2F | PLA2G6 | PLA2R1 | |
| PLAT | PLAUR | PLBD1 | PLCB3 | PLCD3 | PLCXD3 | PLEC | PLEK2 | PLEKHA6 | PLEKHA7 | |
| PLEKHN1 | PLIN3 | PLK1 | PLK4 | PLTP | PML | PMM2 | PNMA3 | PNPLA7 | POC1A | |
| POF1B | POLD4 | POMT1 | PON2 | POU2F3 | PPFIA1 | PPIP5K1 | PPM1D | PPM1E | PPP1CA | |
| PPP1R10 | PPP1R13L | PPP1R14D | PPP1R3B | PPP1R3F | PPP2R2A | PPP2R2B | PPP3CA | PPP3CB | PPP4C | |
| PPP4R1 | PPP4R1L | PPT2 | PRADC1 | PRAF2 | PRC1 | PRDM8 | PRELID2 | PRICKLE3 | PRKDC | |
| PRMT3 | PRMT5 | PROM2 | PRPF40A | PRR11 | PRR5L | PRRC2C | PRRG1 | PRRT1 | PRSS22 | |
| PRSS8 | PSAPL1 | PSD | PSD3 | PSEN1 | PSMA5 | PSMD1 | PSMD14 | PSMD7 | PTBP3 | |
| PTGES | PTK6 | PTPN12 | PTPRR | PTPRU | PTTG1 | PUS7 | PYGB | PYY2 | QDPR | |
| RAB11FIP3 | RAB27B | RAB39A | RAB39B | RAB3C | RAB8A | RABEP1 | RACGAP1 | RAD18 | RAD51 | |
| RAD51AP1 | RAD54L | RAET1E | RAET1L | RAI14 | RALA | RALB | RALBP1 | RANGRF | RAP1B | |
| RASA4 | RASAL1 | RASAL2 | RASD1 | RASEF | RBBP8 | RBM10 | RBM11 | RBM23 | RBM41 | |
| RBM5 | RBM6 | RBM7 | RCAN1 | RCAN2 | RCC1 | RCE1 | RDH10 | REEP2 | REEP3 | |
| RELA | RETSAT | RFC4 | RFWD3 | RFX6 | RGMA | RGS11 | RGS22 | RGS9 | RHBDF1 | |
| RHOC | RHOD | RHPN2 | RIC3 | RIF1 | RIPK2 | RIPK3 | RIPPLY2 | RNA5SP111 | RND2 | |
| RNF122 | RNF141 | RNF146 | RNF149 | RNF167 | RNF168 | RNF169 | RPAIN | RPE | RPL15 | |
| RPL21 | RPL6 | RPS6KA1 | RPS6KA4 | RPSAP52 | RRAS | RRM1 | RRM2 | RSAD1 | RTKN2 | |
| RTN1 | RUNDC3A | RXRB | S100A10 | S100A11 | S100A14 | S100A2 | S100P | SACM1L | SAFB2 | |
| SALL2 | SAMD9 | SARM1 | SAT2 | SBF2 | SCAMP5 | SCG2 | SCG3 | SCG5 | SCML2 | |
| SCN3A | SCN3B | SCNN1A | SDC1 | SDC4 | SDCBP2 | SEC11C | SEC14L2 | SEMA3B | SEMA4B | |
| SEMA7A | SENP3 | SEPHS1 | SERINC1 | SERPINB3 | SERPINB4 | SERPINB5 | SERPINB7 | SERPINB8 | SERPINI1 | |
| SERTAD2 | SESN1 | SETDB2 | SEZ6 | SEZ6L | SFN | SFR1 | SFTA2 | SFTPA2 | SFXN3 | |
| SGMS2 | SGSH | SGSM1 | SGSM2 | SH2D3A | SH2D4A | SH3GL2 | SH3TC2 | SHCBP1 | SHISA7 | |
| SIK2 | SIX1 | SKA1 | SKA3 | SLC12A5 | SLC16A3 | SLC16A5 | SLC1A5 | SLC20A1 | SLC22A17 | |
| SLC22A18AS | SLC23A2 | SLC25A11 | SLC25A24 | SLC25A27 | SLC25A43 | SLC26A11 | SLC29A4 | SLC2A1 | SLC2A11 | |
| SLC30A1 | SLC35A3 | SLC35F2 | SLC38A1 | SLC39A1 | SLC44A1 | SLC52A3 | SLC5A3 | SLC6A14 | SLC6A17 | |
| SLC6A20 | SLC7A14 | SLC7A6OS | SLCO2B1 | SLFN13 | SLK | SMAD3 | SMAGP | SMARCA2 | SMC2 | |
| SMC4 | SMCO2 | SMDT1 | SMIM4 | SMOX | SNAP25 | SNAP91 | SNORD114-2 | SNORD116-26 | SNRNP70 | |
| SNTG1 | SNX12 | SNX7 | SOBP | SOCS2 | SOWAHB | SOWAHC | SP140L | SPAG7 | SPATS2L | |
| SPC24 | SPDEF | SPDL1 | SPIRE2 | SPOCD1 | SPRR1A | SPRR1B | SPRR3 | SPTB | SPTBN2 | |
| SPTLC2 | SPTY2D1 | SQLE | SRPK1 | SRSF12 | SRSF6 | SSBP4 | SSH3 | SSPO | SSRP1 | |
| SSTR2 | ST14 | ST18 | STAMBP | STAT4 | STEAP1 | STIL | STIP1 | STK31 | STK33 | |
| STRN | STS | STX19 | STXBP1 | STXBP5L | STYK1 | SULT2B1 | SUPT4H1 | SVOP | SYP | |
| SYPL2 | SYT14 | SYT4 | SYTL4 | TACSTD2 | TAGLN3 | TALDO1 | TANC1 | TBC1D13 | TBC1D17 | |
| TBC1D2 | TBCC | TBCCD1 | TBKBP1 | TCEA2 | TCEAL1 | TCEAL3 | TCEAL5 | TCF19 | TCF7L2 | |
| TCTA | TCTN1 | TEAD2 | TEAD3 | TEC | TENM1 | TES | TET3 | TFCP2L1 | TFG | |
| TFPI | TFRC | TGFA | THAP7 | THEM4 | TICRR | TIMM22 | TINAGL1 | TJP1 | TK1 | |
| TLE2 | TLL1 | TMBIM1 | TMC7 | TMCC1 | TMEM123 | TMEM139 | TMEM154 | TMEM159 | TMEM171 | |
| TMEM175 | TMEM179 | TMEM241 | TMEM40 | TMEM41A | TMEM59L | TMEM63C | TMEM87A | TMEM87B | TMEM88 | |
| TMEM92 | TMOD3 | TMPRSS4 | TMSB10 | TMTC2 | TMX4 | TNFRSF10A | TNFRSF10B | TNFRSF10D | TNFRSF21 | |
| TNFSF11 | TNIK | TNNT1 | TNR | TNRC6C | TNS4 | TOB1 | TOMM7 | TOP2A | TPM4 | |
| TPX2 | TRAF3IP1 | TRAF7 | TRIM15 | TRIM16 | TRIM29 | TRIM31 | TRIM5 | TRIM59 | TRIP10 | |
| TRIP13 | TSPAN1 | TSPAN15 | TSPAN5 | TSPAN7 | TSPYL1 | TSPYL2 | TSPYL4 | TTK | TTYH3 | |
| TUBA1C | TUBD1 | TUBGCP6 | TXNDC15 | TYMS | UBA6 | UBE2A | UBE2T | UBTF | UCA1 | |
| UCK1 | UEVLD | UHRF1 | UNC119B | UNC13A | UNC13D | UNC5B | UNC79 | UNC80 | UNK | |
| USF2 | USP10 | USP18 | USP20 | USP30 | USP39 | USP51 | USP54 | VAMP2 | VANGL1 | |
| VASP | VDAC1 | VDR | VEGFB | VEZT | VGLL1 | VILL | VIM | VPS35 | VPS4B | |
| VTI1A | VWA5B2 | WDFY1 | WDHD1 | WDR17 | WDR37 | WDR76 | WEE1 | WNK3 | WNT2 | |
| WNT7B | XDH | XKR4 | XPA | XRCC2 | YAP1 | YARS | YEATS2 | YES1 | YWHAZ | |
| YWHAZP3 | ZBTB48 | ZBTB7B | ZCCHC18 | ZCWPW1 | ZDHHC20 | ZDHHC3 | ZDHHC5 | ZDHHC7 | ZDHHC9 | |
| ZER1 | ZFP2 | ZFP3 | ZNF10 | ZNF181 | ZNF185 | ZNF207 | ZNF217 | ZNF248 | ZNF25 | |
| ZNF267 | ZNF32 | ZNF34 | ZNF358 | ZNF367 | ZNF428 | ZNF429 | ZNF483 | ZNF488 | ZNF491 | |
| ZNF540 | ZNF546 | ZNF582 | ZNF583 | ZNF594 | ZNF596 | ZNF641 | ZNF76 | ZNF781 | ZNF79 | |
| ZNF862 | ZSCAN20 | ZSCAN26 | ZW10 | ZWILCH | ZWINT |  |  |  |  | |
