# Supplementary material for: Bioinformatics-based analysis of the relationship between disulfidptosis and prognosis and treatment response in pancreatic cancer
Source: Sci Rep. 2023 Dec 14;13:22218. doi: 10.1038/s41598-023-49752-4 (PMC10721597; doi:10.1038/s41598-023-49752-4)
Supplement: Supplementary file 6 — Supplementary Table S4. [file 41598_2023_49752_MOESM6_ESM.docx]

**Supplementary Table S4 Multifactorial Cox regression analysis of prognosis-related DEGs**

|  |  | |  | |  | |  | |  |
| --- | --- | --- | --- | --- | --- | --- | --- | --- | --- |
| **key genes** | **coef** | **HR** | | **HR.95L** | | **HR.95H** | | **pvalue** | |
| UCA1 | 0.133200764 | 1.142479344 | | 0.951454846 | | 1.371856013 | | 0.153612448 | |
| FNDC3B | 0.835126157 | 2.305104836 | | 1.027256351 | | 5.17252417 | | 0.042849323 | |
| MYBL2 | 0.53004788 | 1.699013655 | | 1.037899262 | | 2.781240439 | | 0.035039986 | |
| NHS | 0.469866938 | 1.599781309 | | 1.048000633 | | 2.442078904 | | 0.029464901 | |
| CCDC15 | 0.505295275 | 1.657474859 | | 0.871516051 | | 3.152234436 | | 0.123399851 | |
