# Supplementary material for: Bioinformatics-based analysis of the relationship between disulfidptosis and prognosis and treatment response in pancreatic cancer
Source: Sci Rep. 2023 Dec 14;13:22218. doi: 10.1038/s41598-023-49752-4 (PMC10721597; doi:10.1038/s41598-023-49752-4)
Supplement: Supplementary file 7 — Supplementary Table S5. [file 41598_2023_49752_MOESM7_ESM.docx]

**Supplementary Table S5 Sensitivity analysis results of 138 chemotherapeutic drugs**

|  |  |  |
| --- | --- | --- |
| **no significant differences** | **more sensitive in low-risk group** | **more sensitive in high-risk group** |
| ABT.263 | ABT.888 | A.443654 |
| AUY922 | AG.014699 | A.770041 |
| AZ628 | AMG.706 | AICAR |
| AZD6244 | AP.24534 | AKT.inhibitor.VIII |
| AZD6482 | AS601245 | AZD.0530 |
| AZD7762 | ATRA | BI.2536 |
| Bexarotene | Axitinib | BIBW2992 |
| BI.D1870 | AZD.2281 | Bicalutamide |
| Bleomycin | AZD8055 | BMS.509744 |
| BMS.754807 | BAY.61.3606 | BMS.536924 |
| Bosutinib | BIRB.0796 | Bortezomib |
| CCT018159 | BMS.708163 | Bryostatin.1 |
| CI.1040 | BX.795 | CGP.082996 |
| Cisplatin | Camptothecin | CGP.60474 |
| Cyclopamine | CCT007093 | CHIR.99021 |
| Cytarabine | CEP.701 | CMK |
| Doxorubicin | DMOG | Dasatinib |
| Embelin | EHT.1864 | Docetaxel |
| Erlotinib | Elesclomol | Epothilone.B |
| Etoposide | GDC.0449 | FTI.277 |
| FH535 | GDC0941 | GNF.2 |
| Gefitinib | IPA.3 | GSK.650394 |
| Gemcitabine | JNK.9L | GW843682X |
| GSK269962A | Lenalidomide | JW.7.52.1 |
| GW.441756 | Metformin | KIN001.135 |
| Imatinib | Methotrexate | Lapatinib |
| JNJ.26854165 | Mitomycin.C | LFM.A13 |
| JNK.Inhibitor.VIII | MK.2206 | NSC.87877 |
| KU.55933 | Nilotinib | NVP.TAE684 |
| MG.132 | NU.7441 | OSI.906 |
| Midostaurin | Nutlin.3a | Paclitaxel |
| MS.275 | NVP.BEZ235 | Parthenolide |
| Obatoclax.Mesylate | PD.0332991 | PD.0325901 |
| PAC.1 | PD.173074 | PHA.665752 |
| Pazopanib | QS11 | PLX4720 |
| PF.02341066 | Roscovitine | Rapamycin |
| PF.4708671 | Salubrinal | RDEA119 |
| PF.562271 | SB590885 | S.Trityl.L.cysteine |
| Pyrimethamine | Temsirolimus | Thapsigargin |
| RO.3306 | TW.37 | VX.680 |
| SB.216763 | Vinblastine | WH.4.023 |
| Shikonin | Vorinostat | WZ.1.84 |
| SL.0101.1 | VX.702 | X17.AAG |
| Sorafenib | ZM.447439 | Z.LLNle.CHO |
| Sunitinib |  |  |
| Tipifarnib |  |  |
| Vinorelbine |  |  |
| WO2009093972 |  |  |
| X681640 |  |  |
| XMD8.85 |  |  |
